# Cell polarity protein Spa2 coordinates Chs2 incorporation at the division site in budding yeast

**DOI:** 10.1371/journal.pgen.1007299

**Published:** 2018-03-30

**Authors:** Magdalena Foltman, Yasmina Filali-Mouncef, Damaso Crespo, Alberto Sanchez-Diaz

**Affiliations:** 1 Instituto de Biomedicina y Biotecnología de Cantabria, Universidad de Cantabria, CSIC, Santander, Spain; 2 Departamento de Biología Molecular, Facultad de Medicina, Universidad de Cantabria, Santander, Spain; 3 Departamento de Anatomía y Biología Celular, Facultad de Medicina, Universidad de Cantabria, Santander, Spain; German Cancer Research Centre (DKFZ), Heidelberg, GERMANY

## Abstract

Deposition of additional plasma membrane and cargoes during cytokinesis in eukaryotic cells must be coordinated with actomyosin ring contraction, plasma membrane ingression and extracellular matrix remodelling. The process by which the secretory pathway promotes specific incorporation of key factors into the cytokinetic machinery is poorly understood. Here, we show that cell polarity protein Spa2 interacts with actomyosin ring components during cytokinesis. Spa2 directly binds to cytokinetic factors Cyk3 and Hof1. The lethal effects of deleting the *SPA2* gene in the absence of either Cyk3 or Hof1 can be suppressed by expression of the hypermorphic allele of the essential chitin synthase II (Chs2), a transmembrane protein transported on secretory vesicles that makes the primary septum during cytokinesis. Spa2 also interacts directly with the chitin synthase Chs2. Interestingly, artificial incorporation of Chs2 into the cytokinetic machinery allows the localisation of Spa2 at the site of division. In addition, increased Spa2 protein levels promote Chs2 incorporation at the site of division and primary septum formation. Our data indicate that Spa2 is recruited to the cleavage site to co-operate with the secretory vesicle system and particular actomyosin ring components to promote the incorporation of Chs2 into the so-called ‘ingression progression complexes’ during cytokinesis in budding yeast.

## Author summary

Eukaryotic cells require division of all their cellular components before finally giving rise to two independent cells in a process named cytokinesis. Eukaryotic cells must build a physical barrier of plasma membrane, which is coupled with the remodelling of the extracellular matrix between the dividing cells. Plasma membrane is transported as part of vesicles by the secretory pathway. In addition, those vesicles carry key cargoes that need to be incorporated into the molecular machinery that promotes cytokinesis, the so-called actomyosin ring. We have previously described that a few components of the actomyosin ring form ‘ingression progression complexes’ or IPCs that coordinate late steps during cytokinesis such as the contraction of the actomyosin ring, ingression of the plasma membrane and extracellular matrix remodelling. Our findings provide new insights into the molecular mechanism by which cells coordinate the secretory pathway, essential for cytokinesis, with the other late cytokinetic steps mentioned above. The cell polarity protein Spa2 plays a key role in that coordination. Spa2 promotes the incorporation into IPCs of the glycosyltransferase Chs2, which is transported on vesicles and essential for extracellular matrix remodelling during cytokinesis in budding yeast. Taking into account that Spa2 protein contains conserved domains present in higher eukaryotes, our work contributes to an understanding of how these domains might play a key role in modulating membrane trafficking and the targeting of specific cargoes to their destination in higher eukaryotes.

## Introduction

Before the end of mitosis, eukaryotic cells need to redirect the secretory machinery towards the site of division to ensure cells deposit additional plasma membrane between the two daughter cells. In addition, secretory vesicles transport key factors to enable cells to perform cytokinesis successfully [1–3]. Although the molecular mechanism is not understood, insertion of membrane and cargoes needs to be highly coordinated with the assembly and contraction of the actomyosin ring, ingression of the plasma membrane and the extracellular matrix remodelling [4–6].

In a process conserved from yeast to mammals, secretory vesicles are transported along actin cables by the type V myosin Myo2. Subsequently, the exocyst complex tethers secretory vesicles to sites of active exocytosis and membrane expansion. The exocyst complex was first identified in the budding yeast *Saccharomyces cerevisiae* and consists of eight subunits: Sec3, Sec5, Sec6, Sec8, Sec10, Sec15, Exo70 and Exo84. Two of them, Sec3 and Exo70, are located to where the secretory vesicle will be targeted and directly bind to PI(4,5)P2, which is situated at the inner leaflet of the plasma membrane. The remaining exocyst components are associated with secretory vesicles [7–9]. It has recently been reported that the exocyst has an open-hand conformation, which explains how the complex tethers secretory vesicles to put them in contact with the plasma membrane [10]. Finally, the association between vesicle and plasma membrane proteins of the SNARE complex (soluble N-ethylmaleimide-sensitive factor attachment protein receptor) leads to the fusion of secretory vesicles with the plasma membrane [7–9, 11, 12]

In *S*. *cerevisiae*, apart from the plasma membrane incorporation during cytokinesis, another essential role for the growth machinery is to deliver key factors such as the chitin synthase Chs2, which lays down a special extracellular matrix layer, the primary septum, between mother and daughter cells. The type V myosin and the exocyst are required for Chs2 localisation to the site of division [13], suggesting a mechanism by which Chs2 is targeted to the cleavage site. However, there must also be a capture mechanism to ensure that Chs2-containing vesicles are incorporated into the cytokinetic machinery while cells are assembling the actomyosin ring prior to the contraction.

To identify novel factors that control the activity of Chs2 at the division site during cytokinesis in budding yeast, we undertook a systematic analysis of regulators of the chitin synthase Chs2 in budding yeast [14]. We previously reported the first part of this work, in which we identified that components of the actomyosin ring, including Chs2, form the so-called ‘ingression progression complexes’ or IPCs. We proposed that the function of these complexes is to coordinate contraction of the actomyosin ring, plasma membrane ingression and primary septum deposition [14]. Here, we describe the role during cytokinesis of the cell polarity protein Spa2, whose molecular details were previously unknown. We show that Spa2 binds to actomyosin ring components during cytokinesis in budding yeast, by interaction between conserved domains within Spa2 and the IPC components Cyk3 and Hof1. Localisation of Spa2 at the cleavage site requires the presence of both IPC components and the growth machinery. We found that Spa2 co-operates with the secretory vesicle system and specific IPC components to promote the incorporation of Chs2 into the cytokinetic machinery.

## Results

### Spa2 interacts with actomyosin ring components during cytokinesis

To understand how cells control the activity of the chitin synthase Chs2 at the division site during cytokinesis, we previously isolated proteins that were able to interact at the same time with Chs2 and one of its regulators, the protein Inn1 [14]. Using mass spectrometry, we identified a specific set of proteins that interact with Inn1-Chs2 complexes at the cleavage site during cell division. Initially we focused on the known core components of the budding yeast actomyosin ring, which we named ‘ingression progression complexes’ (IPCs). The IPCs contain, together with Chs2, the type II myosin Myo1, the IQGAP protein Iqg1, the F-BAR protein Hof1 and the cytokinesis regulators Inn1 and Cyk3 [14].

**Fig 1:**
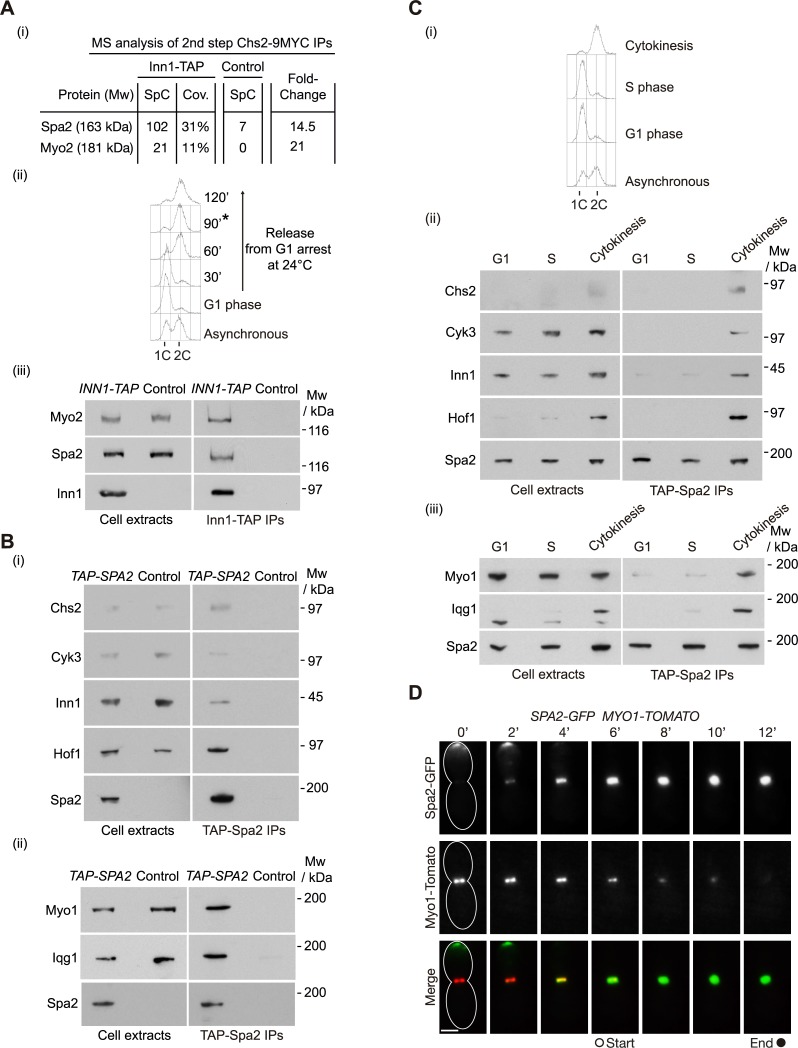
Spa2 interacts with IPCs during cytokinesis. **(A)** *INN1-TAP CHS2-9MYC* (YMF38) and control (YMF79) strains were grown at 24°C in YPRaff medium and synchronised in G1 phase with mating pheromone and then released for 105 minutes. Cell extracts were prepared before the immunoprecipitation of Inn1-TAP (or TAP in control) on IgG-beads. The isolated material was released from the beads by cleavage with TEV protease. Purified material was subjected to immunoprecipitation of Chs2-9MYC before analysis by mass spectrometry [59]. The protein composition of purified fractions was analysed by mass spectrometry. Spectral count number (SpC), percentage sequence coverage (Cov.) and Fold-Change are shown (i). *INN1-TAP MYO2-5FLAG* (YMF969) and control (YMF914) strains were grown at 24°C in YPD medium and synchronised in G1 phase with mating pheromone and then released for 90 minutes before preparing the protein extracts. DNA content was monitored by flow cytometry. The asterisk denotes the time when the sample for making protein extracts was collected (ii) (note that the cytokinesis peak is slightly advanced because the carbon source is different from that in Fig 1A (i)). Cell extracts were prepared and analysed by SDS-PAGE and immunoblotting (iii). **(B)** *TAP-SPA2 CHS2-9MYC* (YMF1302) (i) and *TAP-SPA2 MYO1-5FLAG IQG1-6HA* (YMF1176) (ii), together with the corresponding control strains, were grown at 24°C in YPD medium and synchronised in G1 phase with mating pheromone and then released for 90 minutes. Cell extracts were prepared before immunoprecipitation of Spa2 and detection of the indicated proteins by immunoblotting. **(C)** *TAP-SPA2 CHS2-9MYC* (YMF1302) (ii) and *TAP-SPA2 MYO1-FLAG CYK1-6HA* (YMF1176) (iii) strains were grown at 24°C in YPD medium and synchronised in G1 phase with mating pheromone (G1 sample) or released from G1 block for 30 minutes (S phase sample) or 90 minutes (cytokinesis sample); DNA content was monitored by flow cytometry (i). Cell extracts were then prepared before immunoprecipitating Spa2 on IgG-beads and detecting the indicated proteins by immunoblotting (ii) and (iii). **(D)** *MYO1-Tomato SPA2-GFP (*YMF1256*)* strain was grown at 24°C in YPD and arrested in G1 phase with mating pheromone. Cells were washed and released from G1 arrest into fresh YPD for 30 minutes. Subsequently cells were shifted to Synthetic Complete (SC) medium before being placed on the time-lapse slide to examine the localisation of Myo1 and Spa2 every 2 minutes as cells completed cell division at 24°C (see Methods for details). A z-stack of images was gathered. A two-dimensional projection of the three dimensional data is shown. Scale bar: 2 μm. The grey and black circles denote the timing of the actomyosin ring contraction.

Targeting secretory vesicles to the cleavage site is essential for cytokinesis and occurs from yeast to animal cells. Cells need to incorporate new plasma membrane in order to expand the cell surface and create a physical barrier between mother and daughter cells [1, 4]. In addition, secretory vesicles carry essential cargoes such as the protein Chs2 in budding yeast, a transmembrane protein that is transported to the site of division at the end of the cell cycle [4, 13]. To understand how secretory vesicles carrying Chs2 are incorporated at the site of division and allow Chs2 to be part of the IPCs, we focused our attention on the list of specific proteins that simultaneously interact with Chs2 and Inn1. We identified two proteins by mass spectrometry that could potentially help us to understand the process, since they were previously known to be involved in polarised growth and vesicle transport. The more abundant of the two factors was Spa2 (Fig 1A (i)), which had been suggested to have a role during cytokinesis, although the molecular details were largely unknown. *spa2* mutants show a mild defect in cell separation, and genetic interactions have been described between *SPA2* and other genes involved in cytokinesis, including septin *CDC10* and IPC components *MYO1*, *CYK3* and *HOF1* [15–21]. In addition, we identified myosin type V Myo2 (Fig 1A (i)), which is involved in vesicle transport and delivers essential cargoes such as the chitin synthase Chs2 to the site of division [13]. Together with Spa2, cell polarity proteins Bud6, Pea2 and Bni1 have been described to form the so-called ‘polarisome‘, which plays a role in cell growth [22, 23]. However, we were unable to identify any of the other polarisome components in our mass spectrometry analysis. To confirm that Spa2 and Myo2 were able to interact with the IPC component Inn1, control cells and cells expressing Inn1 fused to TAP were grown at 24°C in the presence of glucose, and cells were synchronised in G1 phase with mating pheromone. Subsequently, we released cells from G1 block and monitored the progression through the cell cycle (Fig 1A (ii)). We pulled down the fusion protein Inn1-TAP from cells released from G1 block for 90 minutes to enrich for cells undergoing cytokinesis and showed that Inn1 co-purified with Spa2 and Myo2 (Fig 1A (iii)). Although the role of Myo2 as a motor protein has been described, the function of Spa2 during cytokinesis was not understood. This prompted us to investigate the Spa2 protein further to understand how secretory vesicles are incorporated into the cleavage site and how the chitin synthase Chs2 is integrated into the IPCs.

To confirm that Spa2 could immunoprecipitate components of the IPCs, we grew *TAP*-*SPA2* and control cells as described above for Fig 1A. We immunoprecipitated TAP-Spa2 and found that Spa2 interacted with all IPC components (Fig 1B (i) and (ii)). To determine precisely when during the cell cycle Spa2 interacts with IPCs, the protein TAP-Spa2 was pulled down from extracts of cells that had been arrested in G1 phase, cells that were going synchronously through S phase, or cells undergoing cytokinesis (Fig 1C (i)). We found that Spa2 only interacted with IPC components at the end of the cell cycle, which suggests a role for Spa2 during cytokinesis (Fig 1C (ii) and (iii)).

To understand the function of Spa2 at the site of division, we first constructed a strain that expressed Spa2-GFP and in which the type II myosin, Myo1, was fused to the red fluorescent protein tandem tomato, Myo1‐Tomato. These cells were released from G1 arrest at 24°C and time-lapse video microscopy was then used to examine when exactly Spa2 localises at the site of division. We found that Spa2 is recruited to the site of division a few minutes before the actomyosin ring starts to contract (Fig 1D), which would suggest precisely the time when CDK-associated kinase activity is inactivated and exactly when Inn1 and Chs2 localise at the division site [24–29]. IPC components appear at the site of division as medial rings and contracted dots, which shows them to be part of the contracting ring. However, Spa2 co-localised with Myo1 at an early stage, but it seems that Spa2 did not share the same localisation with Myo1 later, as it did not appear as a contracted dot (Fig 1D). Spa2 interacts with septins [20], which act as a barrier to compartmentalize proteins around the cleavage site [30]. Therefore, the septin ring might play a role to keep Spa2 at the site of division during contraction. Our data indicate that Spa2 may share a role with IPC components before actomyosin ring contraction starts.

### Spa2-homology domain interacts directly with the SH3-containing proteins Cyk3 and Hof1

To understand the role of Spa2 during cytokinesis, we first determined which of the IPC components were able to interact with Spa2. Although the biological significance was not found, genome-wide screens and genetic analysis showed genetic evidence that *SPA2* could share a role with *CYK3* and *HOF1* [19, 31, 32]. To verify that there is a synthetic lethality between *SPA2* and *CYK3* genes, the meiotic progeny of *spa2Δ cyk3Δ* diploid cells were analysed by tetrad analysis. We confirmed that deletion of the *SPA2* gene in cells lacking the *CYK3* gene led to cell death (S1A (i) Fig).

**Fig 2:**
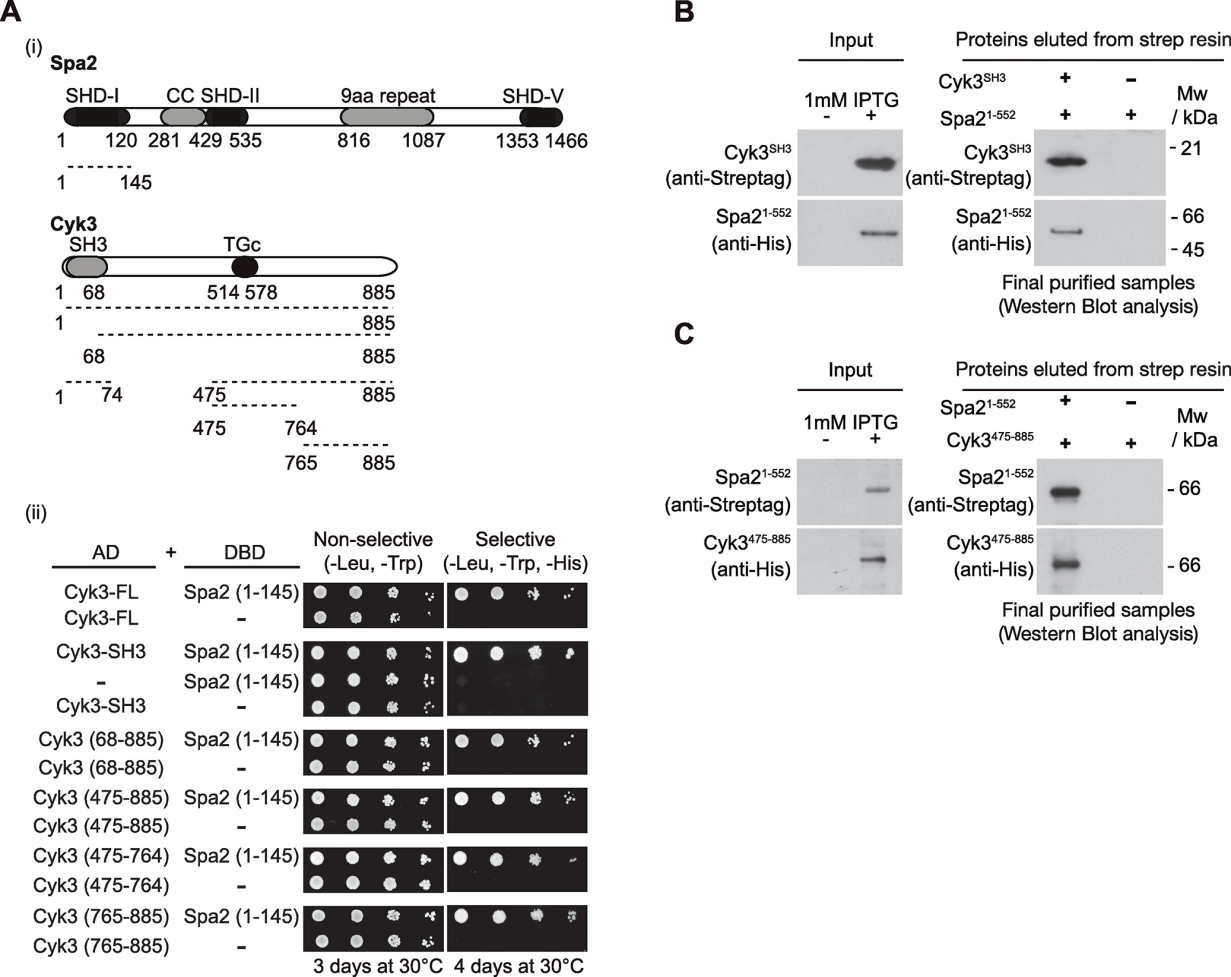
Spa2 interacts directly with multiple domains of Cyk3. **(A)** Diagram of protein structures and yeast two-hybrid interactions between different fragments of Spa2 and tested fragments of Cyk3 (i). Summary of yeast two-hybrid interactions between Spa2-1-145 fragment and different truncations of Cyk3 (ii). **(B)** The SH3 domain of Cyk3 interacts directly with Spa2-1-552. Pairs of *E*. *coli* cell cultures expressing Strep-tag-SH3-Cyk3 and 6His-Spa2-1-552 were mixed and used to purify putative protein complexes via Strep-Tactin Superflow resin. The final purified fractions were analysed by SDS-PAGE and the tagged proteins were detected with anti-Strep or anti-His antibodies. His-tag was only used for protein detection, but not for purification purposes. **(C)** Fragment of Cyk3 containing transglutaminase-like domain interacts directly with Spa2-1-552. Pairs of *E*. *coli* cell cultures expressing Strep-tag- Spa2-1-552 and 6His-Cyk3-475-885 were mixed and used to purify putative protein complexes via Strep-Tactin Superflow resin. The final purified fractions were analysed by SDS-PAGE and the tagged proteins were detected with anti-Strep or anti-His antibodies. His-tag was only used for protein detection, not for purification purposes.

To explore whether Spa2 and Cyk3 can interact physically, two different approaches were taken. First, we used the yeast two-hybrid assay and subsequently studied whether both proteins were able to interact directly in an extract of *E*. *coli* cells. Spa2 protein contains a predicted coil-coiled region and 25 time 9-amino-acid repeats. In addition, Spa2 contains five so-called Spa2 Homology Domains (SHD-I to V), which are conserved domains with the budding yeast Sph1 protein (Fig 2A (i)) [33]. Interestingly, it is the SHD-I that seems to be conserved in higher eukaryotes [33, 34]. It has been reported that SHD-I, SHD-II and SHD-V are the relevant domains for the described dynamics of Spa2 [21, 33, 35, 36]. On the other hand, Cyk3 protein comprises two domains: an N-terminal SH3 domain and a transglutaminase-like domain located in the middle of the protein (Fig 2A (i)). Using a yeast two-hybrid assay, we determined that full-length Cyk3 was able to interact with a fragment of Spa2 that contains the SHD-I (Spa2-1-145) (Fig 2A (ii)). In addition, we found that protein fragments containing the SH3 domain (Cyk3-1-74) and the rest of the protein including the transglutaminase-like domain (Cyk3-68-885), bind to the same fragment of Spa2 (Spa2-1-145) (Fig 2A (ii)). We determined that the Cyk3 transglutaminase-like domain (Cyk3-475-764) and the C-terminal end of Cyk3 (Cyk3-765-885) could interact with Spa2 (Spa2-1-145) as well. Therefore, it seemed that Cyk3 contains multiple sites that bind to Spa2. We found that both Cyk3 domains (SH3 domain and the transglutaminase-like domain) share a function with Spa2, since inactivation of both SH3 (*cyk3-SH3Δ*) and the transglutaminase-like domain (*cyk3-2A*; [14]) induced cell death in *spa2Δ* cells (S1A (ii) and (iii) Fig). This confirms the functional importance of both the SH3 and the transglutaminase-like domains of Cyk3 for Spa2 function.

Next, we generated an *E*. *coli* strain that expressed Strep-tagged SH3 domain of Cyk3 (Strep-tag-Cyk3-SH3) and, in parallel, another strain that produced a truncated version of Spa2 fused to 6His (6His-Spa2-1-552; note that His-tags throughout this work were only used for protein detection, not for purification purposes). We then mixed the cultures and generated a single cell extract containing the SH3 domain of Cyk3, Spa2-1-552 and all native *E*. *coli* proteins. We purified Strep-tag-Cyk3-SH3 from the cell extracts and found that the N-terminal half of Spa2 containing the SHD-I domain co-purified with SH3 domain of Cyk3 (Fig 2B). In addition, using the same experimental design, we expressed a fragment of Cyk3 that contained the transglutaminase-like domain fused to 6His tag (6His-Cyk3-475-885) and, in parallel, a strain that produced the strep-tagged N-terminal truncated version of Spa2 as above (Strep-tag-Spa2-1-552). We purified Strep-tag-Spa2-1-552 from the cell extracts and found that the fragment of Cyk3 containing the transglutaminase-like domain co-purified with Spa2 (Fig 2C). Our data indicate that Cyk3 can bind directly to the N-terminus of Spa2.

**Fig 3:**
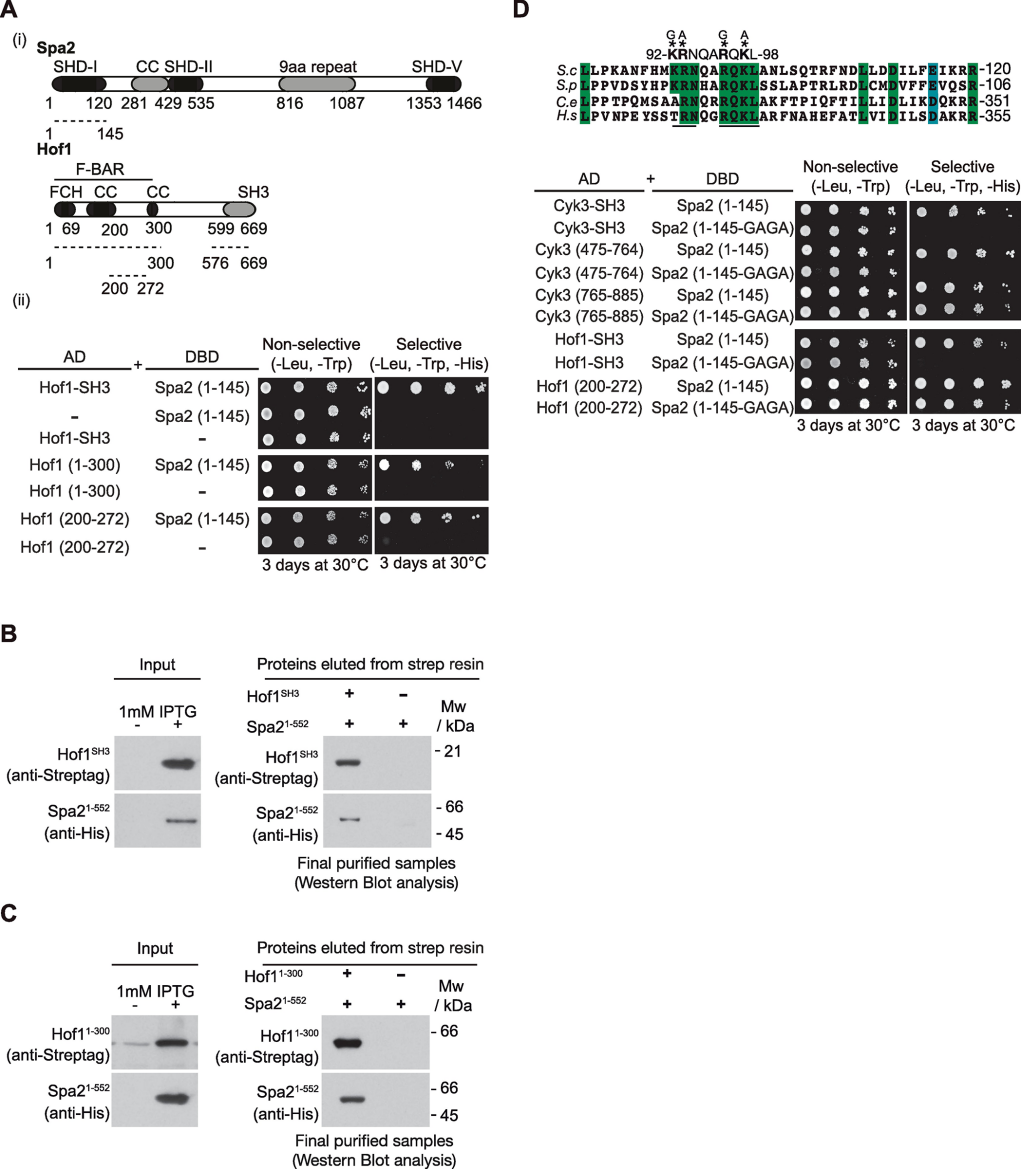
Spa2 interacts directly with multiple domains of Hof1. **(A)** Summary of yeast two-hybrid interactions between the fragments of Spa2-1-145 containing SHD-I and the fragments of Hof1 containing SH3 or F-BAR domains. **(B)** SH3 domain of Hof1 interacts directly with Spa2-1-552. Pairs of *E*. *coli* cell cultures expressing Strep-tag-SH3-Hof1 and 6His-Spa2-1-552 were mixed and used to purify putative protein complexes as in Fig 2B. The final purified fractions were analysed as in Fig 2B. **(C)** The F-BAR domain of Hof1 interacts directly with Spa2-1-552. Pairs of *E*. *coli* cell cultures expressing Strep-tag-Hof1-1-300 and 6His-Spa2-1-552 were mixed and we proceeded as in Fig 2B. **(D)** Schematic alignment and protein sequence comparison of Spa2 (*S*. *cerevisiae*) and its orthologues from *S*. *pombe*, together with SHD domain of *Caenorhabditis elegans* (F14F3.2) and *Homo sapiens* (KIAA0148). The identical residues have been boxed. Conserved Lys and Arg were mutated to Gly and Ala (GAGA). Summary of yeast two-hybrid interactions between a fragment of Spa2-1-145-GAGA containing mutated residues or its wild-type version, and the corresponding fragments of Cyk3 and Hof1.

We confirmed the synthetic lethality between *SPA2* and *HOF1* by tetrad analysis of the meiotic progeny of *spa2Δ hof1Δ* diploid cells (S1B (i) Fig) [19]. As mentioned above in relation to Cyk3, this genetic evidence suggests that Spa2 might share a function with Hof1. In fact, it had been previously shown that Spa2 interacts with Hof1 using fluorescent reporters, although the role of such association was unknown [37]. Hof1 contains an N-terminal F-BAR domain followed by an unstructured region and a C-terminal SH3 domain (Fig 3A). This same structure is observed in other Hof1 orthologues including Cdc15 in *Schizosaccharomyces pombe*, which is involved in actomyosin ring assembly and membrane dynamics [38, 39]. We performed a yeast two-hybrid assay with three different fragments of Hof1 and the first 145 amino acids of Spa2 containing its SHD-I domain (Fig 3A). We found that Spa2 SHD-I interacts with the SH3 domain of Hof1 and with its F-BAR domain, which has been recently crystallised to show that the F-BAR domain of Hof1 is formed of an elongated crescent-shaped dimer [40]. We narrowed down the area within the F-BAR domain that binds to Spa2-1-145. Amino acids 200 to 272 of the F-BAR domain, which corresponds to the convex side of the F-BAR dimer [40], are sufficient to enable such an interaction (Fig 3A). To determine whether Spa2 and Hof1 bind each other directly, we checked, as illustrated in Fig 2B, whether these factors were able to interact in *E*. *coli* extracts expressing the indicated fragments of Spa2 and Hof1. We found that the SH3 and F-BAR domains of Hof1 both bind directly to the N-terminal half of Spa2 containing the SHD-I domain (Fig 3B and 3C).

To detect amino acids that may be relevant in Spa2 interactions, Psi-BLAST searches and secondary structure analysis were performed to show that the amino terminal part of Spa2, which contains the SHD-I, is conserved in other fungal orthologues of Spa2. In addition, it is precisely the SHD-I domain that is conserved in higher eukaryotes [33, 34] (Fig 3D). We identified a stretch of amino acids that is well conserved between fungal and higher eukaryotic orthologues of Spa2 comprising positively charged amino acids, which may be important for Spa2 to bind to its partners. To examine the role of SHD-I, four consecutive conserved basic amino acids were mutated into alanines. A fragment of 145 amino acids containing the wild-type Spa2 SHD-I and the mutated Spa2 SHD-I (Spa2-1-145-GAGA) were used to perform a yeast two-hybrid assay (Fig 3D). We found that the change in these amino acids blocked interactions with SH3 domains of Cyk3 and Hof1, and with the fragment of Cyk3 containing the transglutaminase-like domain of Cyk3 (Cyk3-475-764) (Fig 3D). In contrast, Spa2-1-145-GAGA was able to interact with the C-terminal end of Cyk3 (Cyk3-765-885) and a minimal protein fragment of the F-BAR domain of Hof1 (Hof1-200-272) that we found to interact with Spa2 (Fig 3D).

Taken together, these data indicate that Cyk3 and Hof1 interact directly with Spa2. The SH3 domains of Cyk3 and Hof1, and the transglutaminase-like domain of Cyk3 play a key role with Spa2 SHD-I domain. Positively charged residues within Spa2 are essential for those interactions. In addition, Spa2 SHD-I is able to interact with the F-BAR domain of Hof1 and the C-terminal fragment of Cyk3, but the stretch of positively charged residues that lies within SHD-I seems to be irrelevant for these interactions. Additionally, we found that simultaneous deletions of the SH3 and F-BAR domains of Hof1 induced cell death in *spa2Δ* cells (S1C Fig (iii)), unlike what occurs when single deletions of the SH3 or F-BAR domains of Hof1 are combined with the lack of Spa2 (S1C Fig (i) and (ii)). This confirms the functional importance of both Hof1 domains for Spa2 function (S1C Fig).

### IPC components and the secretory vesicle system share a role in the localisation of Spa2 at the site of division

**Fig 4:**
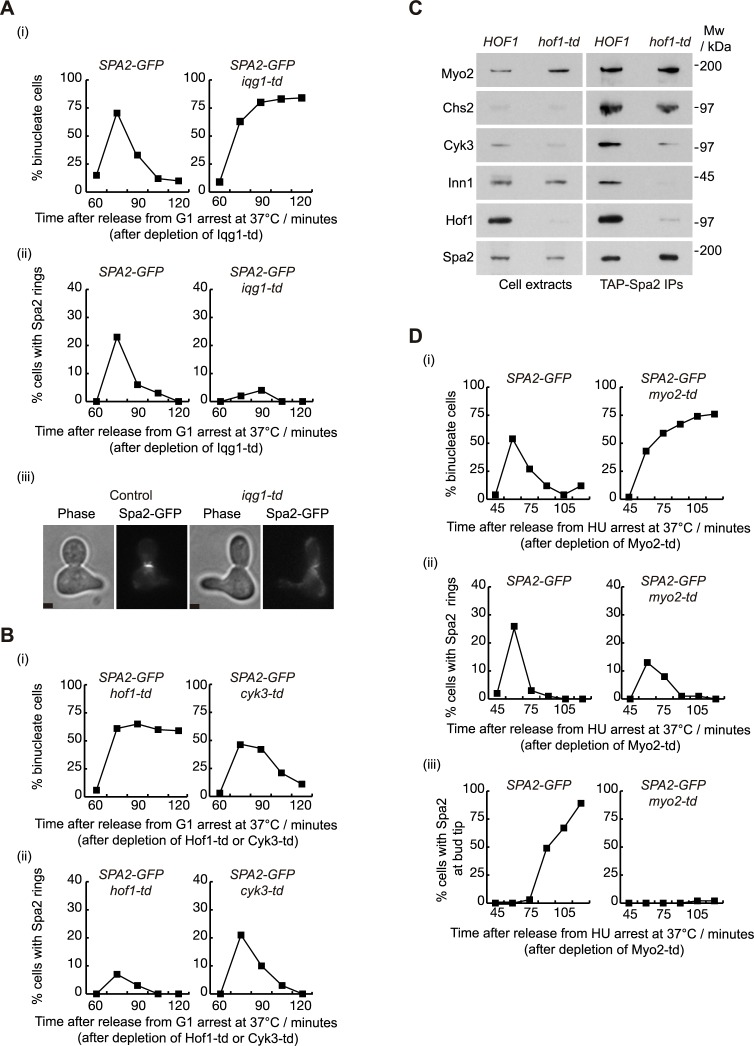
Spa2 protein interacts with secretory vesicle system in the absence of IPC component Hof1. **(A)** *SPA2-GFP* (YMF167) and *SPA2-GFP iqg1-td* (YMF183) strains were arrested in G1 phase at 24°C in YPRaff and then shifted to YPGal at 37°C to deplete Iqg1-td. Subsequently, cells were released to allow progression through the cell cycle. Samples were taken at the indicated times to determine the proportion of binucleate cells (i) and the percentage of cells with single rings of Spa2 at the cleavage site (ii). Example of cell with Spa2-GFP single ring at the bud-neck at 75 minutes is shown (iii). Scale bar: 2 μm. **(B)** *SPA2-GFP hof1-td* (YMF713) and *SPA2-GFP cyk3-td* (YMF1104) strains were grown in YPRaff as in (A) and released from G1 arrest at 37°C after depletion of Hof1-td or Cyk3-td. Samples were taken at the indicated times to determine the proportion of binucleate cells (i) and the percentage of cells with Spa2 rings at the cleavage site (ii). **(C)** *TAP-SPA2* (YMF1301) and *TAP-SPA2 hof1-td* (YMF1299) strains were grown as in (A) and cell extracts were prepared 75 minutes after the release, before the immunoprecipitation of Spa2 and detection of the indicated proteins by immunoblotting. **(D)** *SPA2-GFP* (YMF167) and *SPA2-GFP myo2-td* (YMF716) strains were arrested in G1 phase at 24°C in YPRaff. Cells were then shifted to YPRaff medium containing 0.2 M hydroxyurea to arrested them in early S phase. Cells were allowed to fully grow their buds and were then transferred to YPGal containing 0.2 M hydroxyurea at 37°C in order to deplete Myo2-td. Subsequently, cells were released to allow progression through the cell cycle. Samples were taken at the indicated times to determine the proportion of binucleate cells (i), the percentage of cells with rings of Spa2 at the cleavage site (ii) and the percentage of cells localising Spa2 at the bud tips (iii).

Spa2 interacts with components of the IPCs during cytokinesis (Fig 1). To determine whether the localisation of Spa2 is dependent on IPC components, yeast strains were generated in which the protein Iqg1 or Myo1 was fused to the degron cassette to permit depletion of Iqg1 or Myo1 protein levels [41, 42]. Initially, we grew asynchronous cultures of *iqg1-td SPA2-GFP* and control cells at 24°C before synchronising cells in G1 phase with mating pheromone (Fig 4A; S2 Fig, ‘-td‘ denotes *temperature sensitive degron*). After the induction of Ubr1 E3 ligase and a shift to 37°C to rapidly deplete Iqg1-td protein, cells were released from G1 block at 37°C [42]. We observed that both mutant and control cells progressed to anaphase in a similar manner. Unlike control cells, *iqg1-td SPA2-GFP* accumulated as binucleate cells and contained 4C DNA content as shown by flow cytometry (Fig 4A; S2 Fig), which reflects a failure of cell division. We found that rapid inactivation of Iqg1 prevents localisation of Spa2 at the site of division (Fig 4A (ii)). Similarly, we showed that Myo1 protein is required for Spa2 localisation (S3A Fig). To investigate whether Inn1 promotes Spa2 localisation at the cleavage site, we used the mutant *inn1-td* in which we were able to deplete Inn1 protein levels. We grew *SPA2-GFP inn1-td* and control cells in an identical fashion to that described for Fig 4A and found that Spa2 localisation was independent of Inn1 (S3B Fig).

We determined that Spa2 binds directly to the IPC components Hof1 and Cyk3. Consequently, to explore whether localisation of Spa2 at the cleavage site depends on the interaction with Hof1 and/or Cyk3, we used strains in which we fused the temperature-sensitive degron cassette to the N-terminus of Hof1 or Cyk3 proteins [43]. Cultures of *hof1-td SPA2-GFP* or *cyk3-td SPA2-GFP* strains were grown and synchronised as described for the experiment in Fig 4A. Cells were released after inactivation of Hof1-td or Cyk3-td. Next, we observed that the localisation of Spa2 is indeed defective in *hof1-td* cells, in which binucleate cells accumulated (Fig 4B). In addition, as localisation of Spa2 was only partially altered in *cyk3-td* cells (Fig 4B), we confirmed that Cyk3 protein had been depleted under the restrictive conditions (S4A Fig).

To understand what other factors promote Spa2 localisation at the site of division, *TAP-SPA2 hof1-td* and *TAP-SPA2* control cells were grown as described for the experiment depicted in Fig 4B. After collecting cells going through cytokinesis synchronously in the presence or absence of Hof1, we made cell extracts and immunoprecipitated TAP-Spa2 on IgG-beads to find that in control cells Spa2 co-purified with components of the IPCs, as described above (Fig 1), and the type V myosin Myo2. In Hof1-depleted cells, Spa2 preserved the interaction only with Myo2 and Chs2, however, Spa2 was unable to interact with other IPC components (Fig 4C), which suggests that Spa2 is still able to interact with factors involved in secretory vesicle transport in the absence of any interaction with the IPCs at the site of division. It also suggests that Cyk3 and Inn1 interact with Spa2 through Hof1. To determine whether the Spa2 localisation defect in Hof1-depleted cells reflects a failure in secretory vesicle docking at the site of division or whether it is specific to certain secretory vesicles, we monitored the localisation of the exocyst component Sec8. The exocyst complex drives the delivery of secretory vesicles to the sites of growth during the cell cycle. Before actomyosin ring contraction starts, cells redirect the exocyst to the cleavage site [9]. Using *iqg1-td* cells, we confirmed that Sec8 localisation at the site of division is entirely dependent on the presence of an actomyosin ring (S5A Fig). Subsequently, we carried out similar experiments, inactivating Hof1 instead of Iqg1 (S5B Fig). We found that the exocyst component Sec8 has similar dynamics and localises equally at the site of division in the presence or absence of Hof1, which implies that Hof1 has no role in the docking of the exocyst at the site of division, although it is involved in Spa2 localisation.

Given that motor type V myosin, Myo2, can be pulled down by Spa2 in the absence of Hof1, we analysed the contribution of the secretory vesicle system to the localisation of Spa2 at the site of division. We fused the temperature-sensitive degron cassette to the N-terminus of Myo2 to permit conditional inactivation of the protein. Control and *myo2-td SPA2-GFP* cells were synchronised in G1 phase and then released into S phase in the presence of 0.2 M hydroxyurea, which inhibits ribonucleotide reductase and prevents chromosome replication. Under these conditions, polarised growth continues and buds are able to grow, allowing us to determine the role of Myo2 in the localisation of Spa2 at the site of division during cytokinesis, independently of Myo2 function in bud growth. Once cells had fully grown their buds, Myo2 was depleted by shifting cells to grow at the restrictive temperature of 37°C and expressing the E3 ubiquitin ligase Ubr1. In the absence of functional Myo2, we confirmed that the chitin synthase Chs2 was unable to be targeted to the site of division, as it was previously found using standard temperature sensitive mutants [13] (S6 Fig). We also determined that Spa2 localisation during cell division is only partially defective in the absence of Myo2-driven transport (Fig 4D (i) and (ii)). Spa2 localisation at the tip of new buds in *myo2-td* cells was totally diminished (Fig 4D (iii)), which confirmed that Myo2 was fully inactivated under these experimental conditions. We also confirmed that Myo2 protein levels were depleted soon after cells were transferred to the restrictive conditions (S4B Fig). Taken together, these findings indicate that Spa2 localisation at the site of division partially depends on Hof1 and the secretory vesicle transport, since inactivation of only one of them alone is not enough to block Spa2 localisation at the site of division.

**Fig 5:**
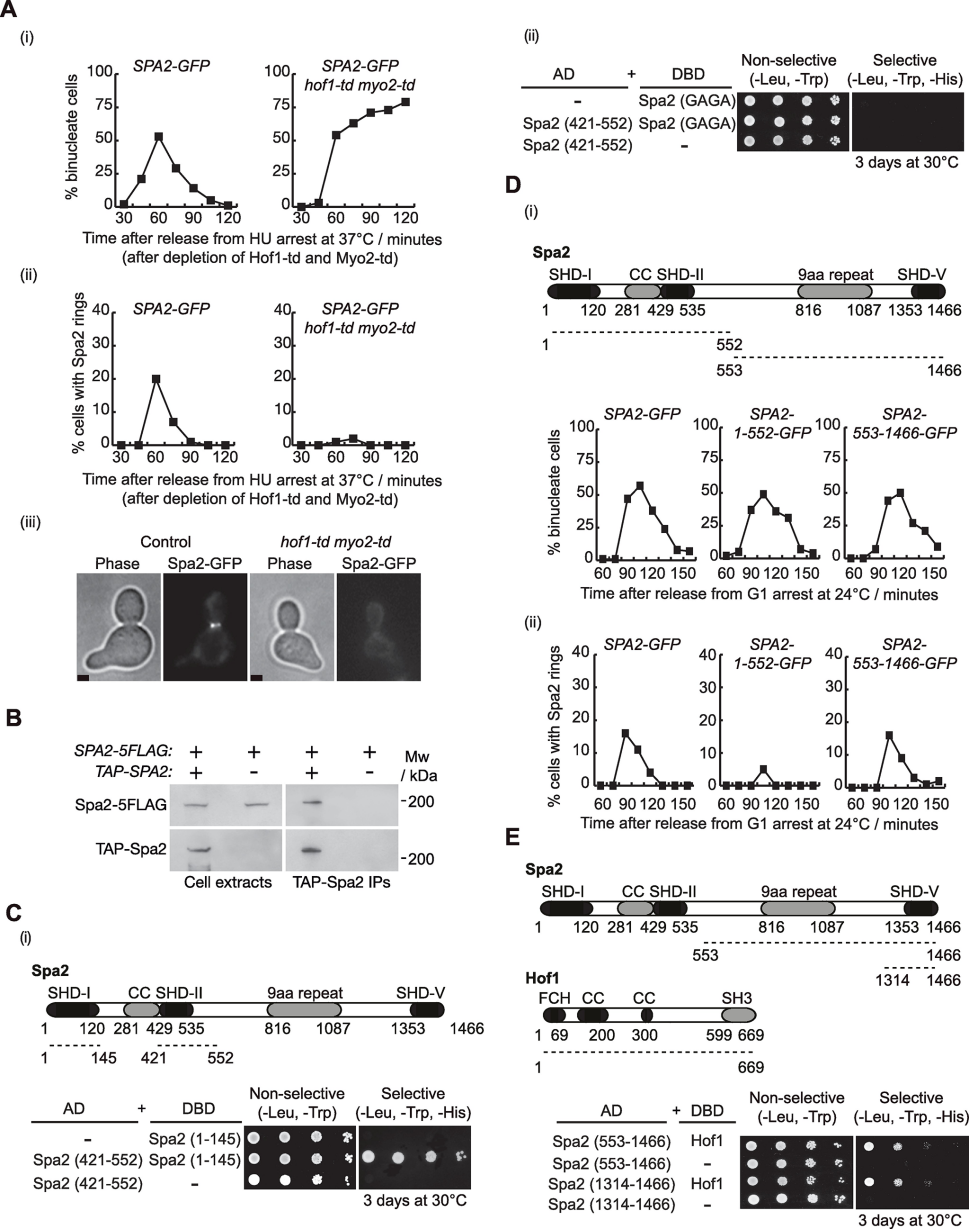
IPC component Hof1 and secretory vesicle transport protein Myo2 both contribute to the localisation of Spa2 at the site of division. **(A)** *SPA2-GFP* (YMF167) and *SPA2-GFP hof1-td myo2-td* (YMF1418) strains were arrested in G1 phase at 24°C in YPRaff and then synchronously shifted to YPRaff medium containing 0.2 M hydroxyurea and arrested in early S phase. Before cells were transferred to YPGal containing 0.2 M hydroxyurea at 37°C in order to deplete Hof1-td and Myo2-td, they were allowed to grow their buds. Subsequently, cells were released to allow progression through the cell cycle. Samples were taken at the indicated times to determine the proportion of binucleate cells (i) and the percentage of cells with Spa2 rings at the cleavage site (ii). An example of a cell with Spa2-GFP ring at the bud-neck at 60 minutes is shown (iii). Scale bar: 2μm. **(B)** Indicated diploid strains *TAP-SPA2/SPA2-5FLAG* (YMF1448) and *SPA2/SPA-5FLAG* (YMF1449) were grown asynchronously at 24°C in YPD medium and cell extracts were prepared before immunoprecipitation of TAP-Spa2 and detection of the indicated proteins by immunoblotting. **(C)** Summary of yeast two-hybrid interactions between the different fragments of Spa2, one containing SHD-I region (Spa2-1-145) and another one containing SHD-II (Spa2-421-552). **(D)** *SPA2-GFP* (YMF117), Δ*C-SPA2-GFP* (YMF967, Spa2-1-552-GFP) and Δ*N-SPA2-GFP* (YMF1023, Spa2-553-1466-GFP) strains were arrested in G1 phase at 24°C in YPD and then released from G1 arrest to allow progression through the cell cycle. Samples were taken at the indicated times to determine the proportion of binucleate cells (i) and the percentage of cells with rings of Spa2 or its truncations at the cleavage site (ii). **(E)** Summary of yeast two-hybrid interactions between the C-terminal fragments of Spa2 and Hof1.

To determine whether Hof1 and Myo2 share a role in the localisation of Spa2, we grew control and *hof1-td myo2-td* cells under the same conditions as described for Fig 4C and found the localisation of Spa2 to be completely dependent on both Hof1 and Myo2 proteins (Fig 5A; S7 Fig). There appear to be two populations of Spa2, one that binds to the IPCs via Hof1, and another that interacts with the secretory vesicle transport system. We speculated that these two populations might associate through Spa2 itself. To investigate whether Spa2 might interact with itself, a diploid yeast strain was generated in which one *SPA2* gene expressed TAP-Spa2, while the other expressed Spa2-5FLAG. These diploid cells, together with diploid cells that lacked the expression of *TAP-SPA2* as control, were grown asynchronously. Yeast protein extracts were made and TAP-Spa2 was subsequently pulled down on IgG beads. We showed that TAP-Spa2 interacted with Spa2-5FLAG (Fig 5B), suggesting that Spa2 might be able to form dimers. Using the yeast two-hybrid assay, we found that the amino-terminus of Spa2 containing the SHD-I domain was able to interact with a fragment of Spa2 comprising the SHD-II, which would leave open such a possibility (Fig 5C (i)). Furthermore, we found that both fragments were unable to interact if the SHD-I domain was mutated to eliminate positively charged amino acids (Fig 5C (ii)). Taken together, these experiments indicate that Hof1 and Myo2 share a role in the localisation of Spa2 and that Spa2 proteins might form dimers.

To determine whether Spa2 domains control localisation of Spa2 at the site of division in a different manner, the chromosomal *SPA2* locus was modified so that cells expressed either Spa2-1-552-GFP (containing the SHD-I and SHD-II domains) or Spa2-553-1466-GFP (comprising multiple 9-aminoacid repeats and the SHD-V domain) under the control of *SPA2* promoter. We grew *SPA2-GFP* cells in parallel with cells expressing either the N-terminal (1-552-GFP) or C-terminal fragment of Spa2 (553-1466-GFP). Cells were arrested in G1 phase and subsequently released synchronously to monitor the localisation of each construct at the site of division (Fig 5D; S8 Fig). We found that the localisation of the N-terminal half of Spa2 (1-552-GFP) was clearly defective (Fig 5D (ii); S8A Fig). Expression of Spa2 truncations was confirmed using immunoblotting analysis (S8B Fig). This suggests that the C-terminus of Spa2 is required for Spa2 to localise at the site of division. To investigate whether Spa2-553-1466 can interact with Hof1 in the same manner as shown above for Spa2-1-552 (Fig 2), we used the yeast two-hybrid analysis to show that, indeed, the fragment of Spa2 containing amino acids 553–1466 interacts with Hof1 (Fig 5E). We found that the SHD-V domain is sufficient to bind to Hof1 (Fig 5E). Taken together, these results indicate that Spa2 domains are able to interact with the IPC component Hof1. The C-terminal of Spa2 protein seems to be more relevant in the recruitment of Spa2 to the site of division.

### Spa2 protein interacts directly with the chitin synthase Chs2

**Fig 6:**
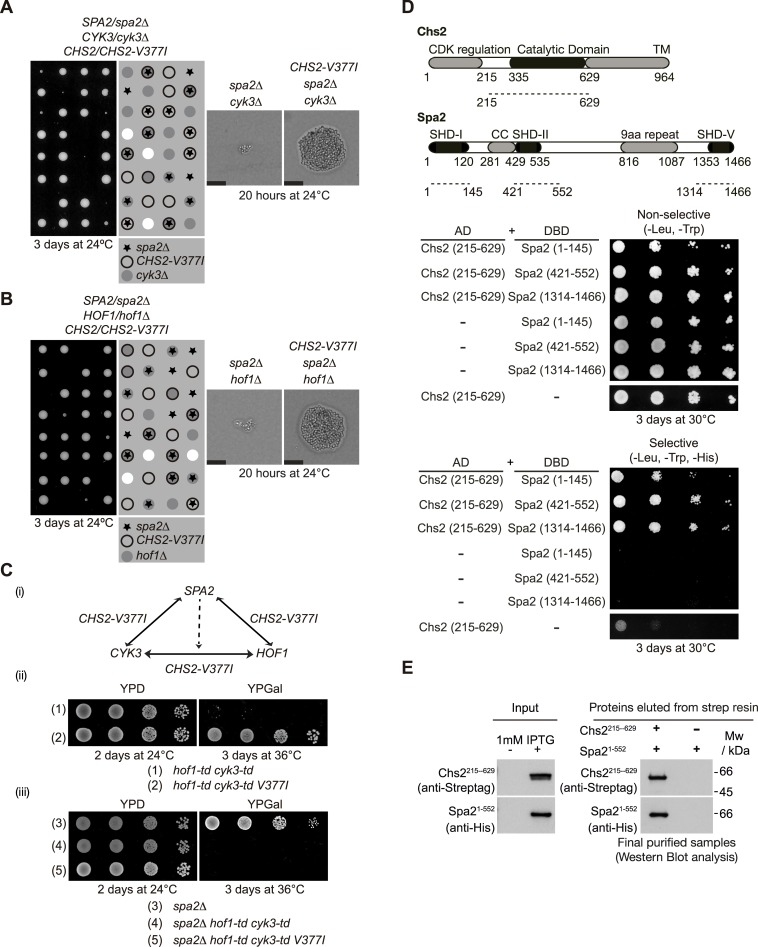
Spa2 interacts directly with the chitin synthase Chs2. **(A)** Tetrad analysis of the meiotic progeny from the indicated diploid strain (YMF741) shows that *CHS2-V377I* allows *spa2*Δ *cyk3*Δ cells to grow. Spores of the indicated genotypes were grown for 20 hours on YPD plates at 24°C. Scale bar: 20 μm. **(B)** Tetrad analysis of the meiotic progeny from the indicated diploid strain (YMF866) shows that *CHS2-V377I* allows *spa2*Δ *hof1*Δ cells to grow. Spores of the indicated genotypes were grown for 20 hours on YPD plates at 24°C. Scale bar: 20 μm. **(C)** Scheme representing the genetical relationship between Cyk3, Hof1, Spa2 and Chs2-V377I proteins (i). Serial dilutions of strains YMF140 (1) and YMF1307 (2) were plated on YPD medium or YPGal medium and incubated as indicated (ii). Serial dilutions of strains YMF759 (3), YMF1401 (4) and YMF1399 (5) were plated on YPD medium or YPGal medium and incubated as indicated (iii). **(D)** Summary of yeast two-hybrid interactions between the different fragments of Spa2 and the Chs2. Truncated allele of Chs2 containing the catalytic domain (Chs2-215-629) was used to show that this region of Chs2 (Chs2-215-629) interacts in a yeast two-hybrid assay with Spa2 via different SHD-containing fragments. **(E)** Pairs of *E*. *coli* cell cultures expressing Strep-tag-Chs2-215-629 and 6His-Spa2-1-552 were mixed and used to purify putative protein complexes via Strep-Tactin Superflow resin. The final purified fractions were analysed by SDS-PAGE and the tagged proteins were detected with anti-Strep or anti-His antibodies. His-tag was only used for protein detection, not for purification purposes.

The protein Spa2 is essential in the absence of either Cyk3 or Hof1 (S1 Fig) [19, 31, 32]. We proposed that understanding why Spa2 becomes essential in *spa2Δ cyk3Δ* cells and *spa2Δ hof1Δ* cells could reveal the molecular details of Spa2 role during cytokinesis. We and others have reported that Cyk3 and Hof1 proteins regulate the function of the chitin synthase Chs2, which lays down the primary septum between mother and daughter cells during cytokinesis [14, 28, 44–46]. To determine whether Spa2, together with Hof1 and/or Cyk3, has a role related to Chs2 function, we performed genetic analyses in which we tried to rescue defects associated with the double mutants *spa2Δ cyk3Δ* and *spa2Δ hof1Δ*. We constructed a diploid strain lacking one copy of *CYK3*, one copy of *SPA2* and harbouring a hypermorphic allele of *CHS2*, which has enhanced chitin synthase activity [44]. The meiotic progeny was then analysed by tetrad analysis (Fig 6A). We found that hypermorphic Chs2 (*CHS2-V377I*) suppressed the growth defect at 24°C caused by the lack of the Cyk3 and Spa2 proteins (Fig 6A). Following the same strategy we also showed that *CHS2-V377I* rescues defects associated with *spa2Δ hof1Δ* cells at 24°C (Fig 6B).

Therefore, we found that growth defects associated with the lack of Cyk3 and Spa2, or Hof1 and Spa2 could be rescued by the hypermorphic allele of *CHS2* (Fig 6C (i)). To complete the genetic analysis of *CYK3*, *HOF1* and *SPA2*, we showed that growth defects associated with double degron *hof1-td cyk3-td* strains under restrictive conditions were rescued by the hypermorphic allele *CHS2-V377I* (Fig 6C (ii)). It seems that *HOF1*, *CYK3* and *SPA2* form a network of factors whose defects associated with the lack of function in pairs are always rescued by *CHS2-V377I* (Fig 6C (i)). Finally, we determined that *SPA2* becomes essential for *CHS2-V377I* to rescue the absence of Hof1 and Cyk3, since *spa2Δ hof1-td cyk3-td CHS2-V377I* cells were unable to grow (Fig 6C (ii) and (iii), compare strains (2) and (5)). Taken together, these genetic analyses indicate that Spa2 plays a role related to the chitin synthase Chs2 during cytokinesis.

To look in greater detail at the Spa2-Chs2 functional relationship, we examined whether Spa2 physically interacts with Chs2. First we used the yeast two-hybrid assay to show that a fragment of Chs2 comprising its catalytic domain (Chs2-215-629) interacts with Spa2 truncations containing the SHD-I, SHD-II or SHD-V domains (Fig 6D). Subsequently, we used *E*. *coli* cells to express 6His-Spa2-1-552, which contains two of the domains that we found to interact with Chs2 in the yeast two-hybrid assay (SHD-I, SHD-II), in parallel with another strain that expressed Strep-tag-Chs2-215-629 that comprises its catalytic domain. We found that both proteins interacted directly (Fig 6E), which supports the hypothesis that Spa2 function during cytokinesis is related to chitin synthase Chs2.

### Hof1 and Cyk3 co-ordinately promote initial incorporation of Spa2 at the site of division

Spa2 localises at the site of division a few minutes before the actomyosin ring contraction starts (Fig 1D). In addition, we found that inactivation of the IPC components Hof1 or Cyk3 alters the localisation of Spa2 and that the defect is clearly more severe when Hof1 is not present (Fig 4B). Furthermore, our genetic analysis showed that growth defects associated with the lack of Hof1/Spa2, Cyk3/Spa2 and Hof1/Cyk3 are rescued by the expression of a hypermorphic allele of *CHS2*. Finally, we found that Spa2 directly binds to Hof1, Cyk3 and, independently of Hof1, to Chs2 when it is being transported via motor protein Myo2. Therefore, we hypothesised that Hof1 and Cyk3 contribute to the docking of Chs2-containing vesicles at the site of division, for which Spa2 could play a role.

**Fig 7:**
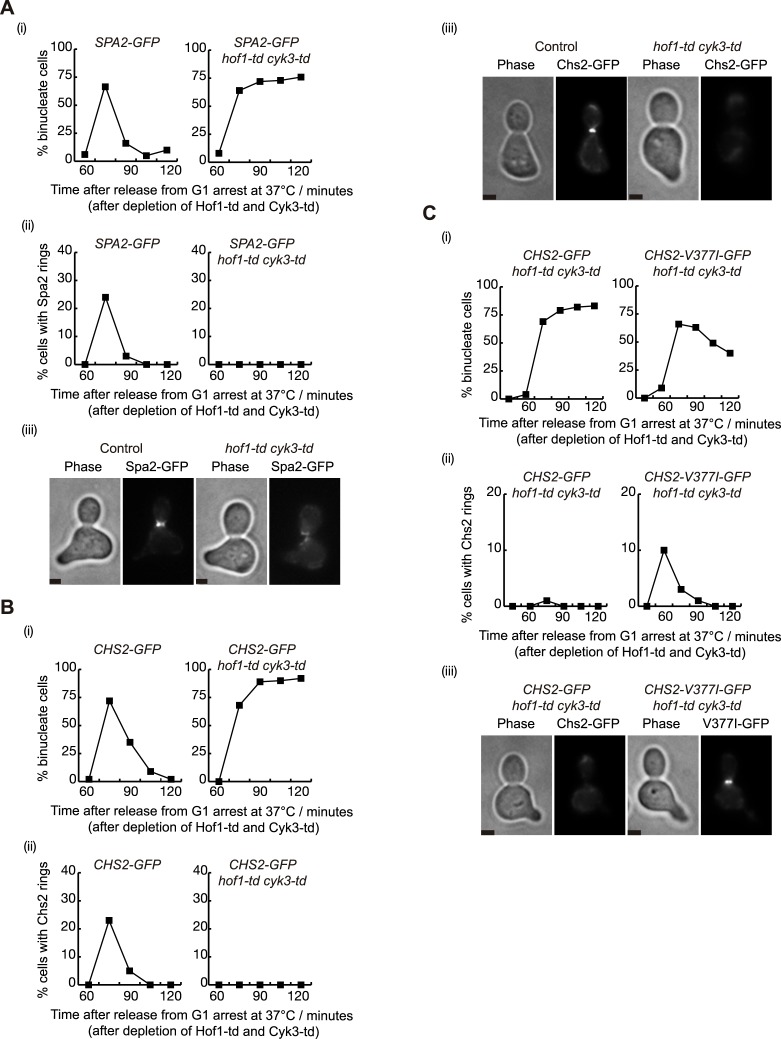
Spa2 localisation at the site of division requires the presence of Hof1 and Cyk3. **(A)** *SPA2-GFP* (YMF167) and *SPA2-GFP hof1-td cyk3-td* (YMF1088) strains were arrested in G1 phase at 24°C in YPRaff and then shifted to YPGal at 37°C to deplete Hof1-td and Cyk3-td simultaneously. Subsequently, cells were released to allow progression through the cell cycle. Samples were taken at the indicated times to determine the proportion of binucleate cells (i) and the percentage of cells with rings of Spa2 at the cleavage site (ii). Example of cell with Spa2-GFP ring at the bud-neck is shown for the 75 minute time-point (iii). Scale bar: 2 μm. **(B)** *CHS2-GFP* (YMF330) and *CHS2-GFP hof1-td cyk3-td* (YMF1076) strains were grown in YPRaff as in (A). Samples were taken at the indicated times to determine the proportion of binucleate cells (i) and the percentage of cells with rings of Chs2 at the cleavage site (ii). Example of cell with Chs2-GFP ring at the bud-neck at 75 minutes (iii). Scale bar: 2 μm. **(C)** *CHS2-GFP hof1-td cyk3-td* (YMF1076) and *CHS2-V377I-GFP hof1-td cyk3-td* (YMF1329) strains were grown in YPRaff as in (A). Samples were taken at the indicated times to determine the proportion of binucleate cells (i) and the percentage of cells with rings of Chs2 or Chs2-V377I at the cleavage site (ii). Example of cell with Chs2-V377I-GFP ring at the bud-neck at 60 minutes (iii). Scale bar: 2μm.

To test this hypothesis we first determined whether both Hof1 and Cyk3 shared a coordinated role in the localisation of Spa2 at the cleavage site. Cultures of *SPA2-GFP* and *hof1-td cyk3-td SPA2-GFP* cells were grown at 24°C and cells were synchronised in G1 phase with mating pheromone, before rapidly inactivating Hof1 and Cyk3 at 37°C. Upon release from G1 arrest at 37°C, *hof1-td cyk3-td* cells completed mitosis but were unable to divide in the same way as control cells (Fig 7A (i)). Localisation of Spa2 at the site of division was not observed in the absence of Hof1 and Cyk3 (Fig 7A (ii)), despite the presence of Spa2 protein in mutant cells (S9A Fig).

Since we have previously described that Chs2 localisation is defective in Hof1-depleted cells [14], and Hof1, Cyk3 and Spa2 seem functionally connected to Chs2, we aimed to confirm whether the localisation of Chs2 at the site of division was co-ordinately dependent on both Hof1 and Cyk3. We grew *hof1-td cyk3-td CHS2-GFP* and control cells as described above for Fig 7A. Chs2 protein levels were confirmed in control and mutant cells (S9B Fig). Interestingly, we found that localisation of Chs2 was completely defective if Hof1 and Cyk3 had been previously depleted (Fig 7B and S9C Fig). This was unexpected since the hypermorphic allele *CHS2-V377I* rescues *hof1-td cyk3-td* cells (Fig 6C (ii)).

On the other hand, we have previously shown that Chs2-V377I is constitutively active *in vitro* [44]. We anticipated that Chs2-V377I should localise at the site of division in order to rescue any defects associated with *hof1-td cyk3-td* cells. We investigated this by growing *hof1-td cyk3-td CHS2-V377I*-GFP and *hof1-td cyk3-td CHS2*-GFP cells as described for Fig 7B. Indeed, Chs2-V377I partially recovered Chs2 localisation at the site of division (Fig 7C (ii) and (iii)), as was reflected by the partial rescue of the cell division defect associated with *hof1-td cyk3-td* cells (Fig 7C (i)). Therefore, we concluded that structural changes promoted by the change in amino acid V377 were enough to drive Chs2 localisation during cytokinesis.

### Artificial recruitment of the chitin synthase Chs2 to the actomyosin ring promotes the localisation of Spa2 at the site of division

**Fig 8:**
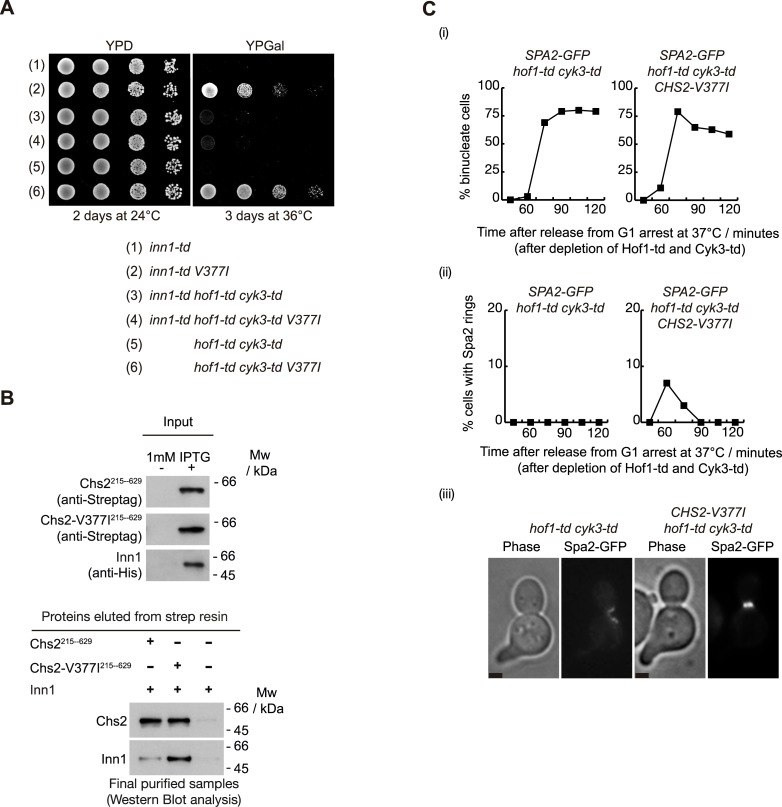
Higher-affinity binding of Chs2-V377I to Inn1 promotes recruitment of Spa2 at the site of division in the absence of Hof1 and Cyk3. **(A)** Serial dilutions of strains YASD522 (1), YMF1375 (2), YMF1394 (3), YMF1370 (4), YMF140 (5) and YMF1307 (6) were plated on YPD medium or YPGal medium and incubated as indicated. **(B)** Chs2-V377I has a higher affinity for binding to Inn1. Pairs of *E*. *coli* cell cultures expressing either Strep-tag-Chs2-215-629 or Strep-tag-Chs2-215-629-V377I were mixed with cultures expressing 6His-Inn1 and used to purify putative protein complexes via Strep-Tactin Superflow resin. The final purified fractions were analysed by SDS-PAGE and the tagged proteins were detected with anti-Strep or anti-His antibodies. His-tag was only used for protein detection, but not for purification purposes. **(C)** *SPA2-GFP CHS2 hof1-td cyk3-td* (YMF1088) and *SPA2-GFP CHS2-V377I hof1-td cyk3-td* (YMF1403) strains were arrested in G1 phase at 24°C in YPRaff and then shifted to YPGal at 37°C to deplete Hof1-td and Cyk3-td simultaneously. Subsequently, cells were released to allow progression through the cell cycle. Samples were taken at the indicated times to determine the proportion of binucleate cells (i) and the percentage of cells with rings of Spa2 at the cleavage site (ii). Example of cell with Spa2-GFP ring at the bud-neck at 60 minutes (iii). Scale bar: 2 μm.

We have previously described that the Inn1 protein contributes to the localisation of Chs2 at the actomyosin ring [14] and that the hypermorphic allele *CHS2-V377I* rescues the defect in Inn1-depleted cells [44] (Fig 8A, compare (1) and (2)). To determine whether Inn1 is a key protein in the rescue of *hof1-td cyk3-td* cells by *CHS2-V377I*, we generated a strain that contained Hof1, Cyk3 and Inn1 fused to the temperature-sensitive degron cassette in order to deplete levels of all three proteins. We performed a growth assay and found that *CHS2-V377I* was no longer able to rescue *hof1-td cyk3-td* cells in the absence of Inn1 (Fig 8A, compare (4) and (6)). Our data would suggest that the three key factors that enable Chs2-V377I to dock at the actomyosin ring are Hof1, Cyk3 and Inn1.

Therefore, Inn1 might explain why *CHS2-V377I* localises at the site of division and rescues *hof1-td cyk3-td* cells. Inn1 directly binds to Chs2 [14]. We hypothesised that the association between Chs2-V377I and Inn1 might be stronger than between the wild-type Chs2 and Inn1, which would explain why *CHS2-V377I* rescues the defect in these cells. We generated *E*. *coli* strains that produced Strep-tag-Chs2-215-629, Strep-tag-Chs2-V377I-215-629 and 6His-tagged-Inn1. We then mixed the cultures in pairs (Strep-tag-Chs2-215-629/6His-tagged-Inn1, Strep-tag-Chs2-V377I-215-629/6His-tagged-Inn1 and empty vector control/6His-tagged-Inn1) and generated a single cell extract containing Chs2/Inn1, Chs2-V377I/Inn1 or no tagged protein/Inn1, together with all native bacterial proteins (Fig 8B). Next we purified the same amount of Strep-tag-Chs2 or Strep-tag-Chs2-V377I from the cell extracts, and subsequently determined that the amount of Inn1 protein purified with Strep-tag-Chs2-V377I was almost three times that of the Inn1 protein isolated from the cell extract with Strep-tag-Chs2 (Fig 8B). These findings suggest that Chs2-V377I interacts more strongly with Inn1, which must be enough to be incorporated in the IPCs in the absence of Hof1 and Cyk3.

The proposed model for Spa2 localisation at the cleavage site suggests dual mechanisms: Spa2 interacts with IPC components and with members of the secretory vesicle transport. To test whether Spa2 localisation at the site of division is recovered in cells in which Hof1 and Cyk3 proteins had been depleted, we investigated whether artificial recruitment of Chs2 (via Chs2-V377I) to the actomyosin ring was sufficient to induce Spa2 localisation. We grew *SPA2-GFP CHS2 hof1-td cyk3-td* cells and *SPA2-GFP CHS2-V377I hof1-td cyk3-td* cells as described above for Fig 7C. We observed that Spa2 protein can partially localise at the site of division when cells are expressing the hypermorphic allele of *CHS2* in the absence of Hof1 and Cyk3 (Fig 8C). Our findings would suggest that part of the Spa2 protein population might require the arrival of Chs2-containing vesicles at the site of division in order to finally localise before the actomyosin ring contraction starts and successful cytokinesis takes place.

### Increased Spa2 protein levels promote Chs2 incorporation at the site of division

**Fig 9:**
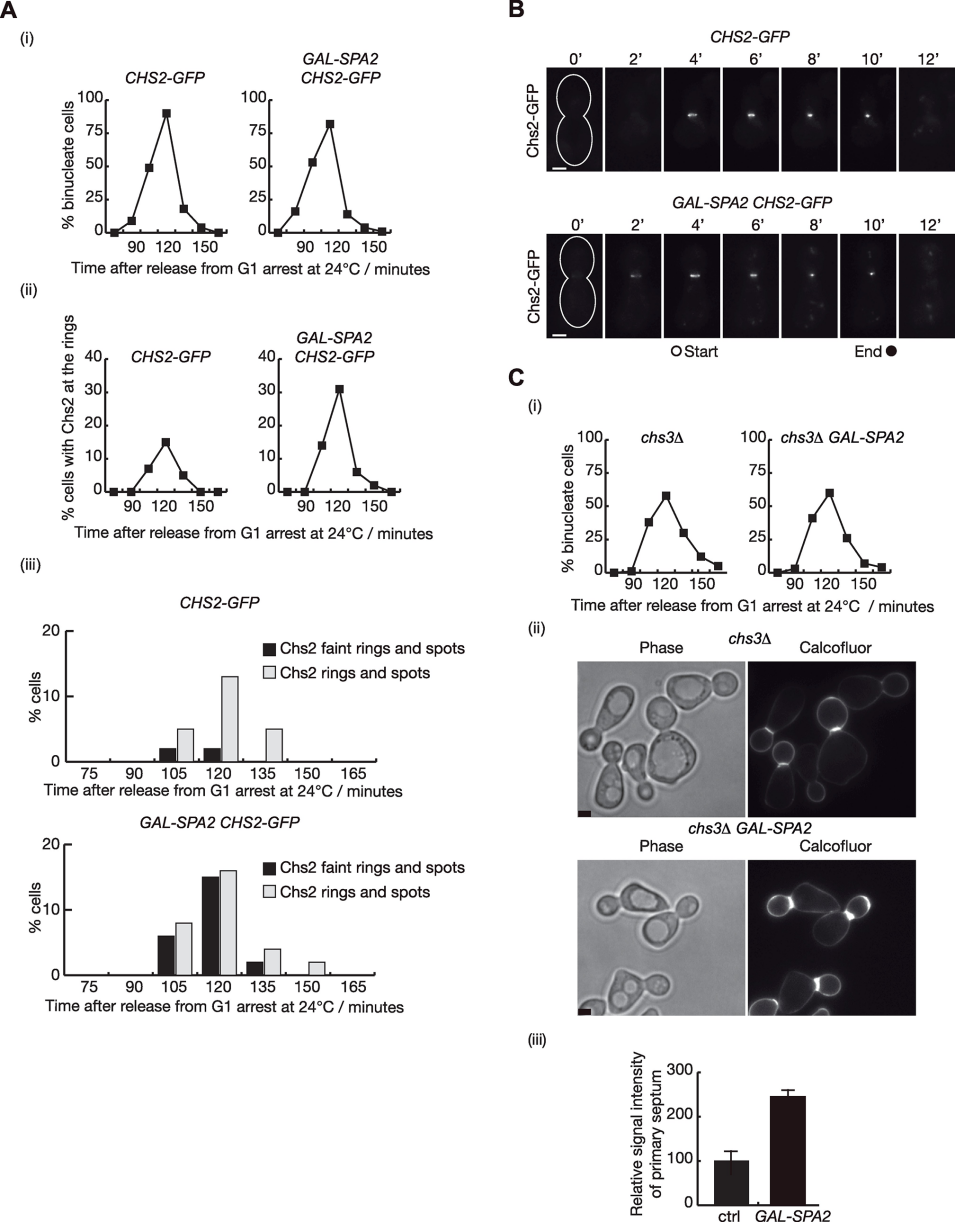
Spa2 coordinates delivery of Chs2 to the site of division. **(A)** *CHS2-GFP* (YASD819) and *CHS2-GFP GAL-SPA2* (YMF1660) strains were arrested in G1 phase at 24°C in YPRaff and then shifted to YPGal at 24°C to induce expression of Spa2. Subsequently, cells were released from G1 block to allow progression through the cell cycle. Samples were taken at the indicated times to determine the proportion of binucleate cells (i) and the percentage of cells with Chs2 rings at the cleavage site (ii) and (iii). **(B)** *CHS2-GFP SPC42-EQFP* (YAD380) strain together with *CHS2-GFP GAL-SPA2* (YMF1660) were grown as in Fig 8A. 75 minutes after the release from G1 block cells were shifted to SC medium before being placed on the time-lapse slide to examine the localisation of Chs2 every 2 minutes as cells completed cell division at 24°C (see Methods for details). A z-stack of images was gathered. A two-dimensional projection of the three-dimensional data is shown. Scale bar: 2 μm. The grey and black circles denote the timing of the actomyosin ring contraction. **(C)** *chs3Δ* control (YMF505) and *chs3Δ GAL-SPA2* (YMF1534) cells were grown in YPRaff medium at 24°C and synchronised in G1 with alpha factor. Subsequently, cells were released in YPGal for 135 minutes from G1 block in the presence of calcofluor to visualise primary septum deposition. Samples were taken at the indicated times to determine the proportion of binucleate cells (i) and 100 cells with primary septum for each sample were examined. Examples of live cells grown with calcofluor are shown in (ii). Scale bar: 2 μm. The relative signal intensity of primary septum was measured for 100 cells and compared with control cells, whose signal intensity was set to 100% (iii).

To investigate whether overexpression of Spa2 might increase Chs2 incorporation at the site of division, strains overexpressing *SPA2*, together with control, were grown at 24°C and cells were synchronised in G1 phase. Cells were then released from G1 arrest into medium containing galactose to allow overexpression of Spa2. Subsequently, samples were used to examine the incorporation of Chs2-GFP at the division site by fluorescence microscopy (Fig 9A). Cells overexpressing Spa2 increased the number of Chs2-GFP rings (Fig 9A (ii)), mainly due to the increase of rings with a fainter fluorescent signal associated with Chs2-GFP (Fig 9A (iii)). Normally, Chs2-GFP signal at the site of division initiates as a faint ring that turns into a ring with a stronger fluorescent signal before actomyosin ring contraction starts. As cell cycle progression seemed to be similar in control and *GAL-SPA2* cells (Fig 9A (i)), we hypothesised that those faint rings might indicate that there is slightly more Chs2 protein at the site of division in cells overexpressing Spa2, and Chs2-GFP signal passed the threshold of detection under fluorescent light. To confirm our hypothesis, time-lapse video microscopy was used to examine Chs2-GFP localisation (Fig 9B). Cells were grown in the same way as described above for Fig 9A. 75 minutes after the release from G1 block, cells were shifted to Synthetic Complete (SC) medium and subsequently placed in a time-lapse slide to examine the localisation of Chs2 at 24°C every 2 minutes (see Methods). Both control and *GAL-SPA2* cells were treated in an identical fashion, since the cultures were mixed before the cells were transferred to the time-lapse slide (the control cells expressed Spc42-eQFP and could therefore be distinguished from *GAL-SPA2* cells). Twenty movies each were examined for control and *GAL-SPA2 CHS2-GFP* cells. We were able to detect Chs2-GFP signal 2 minutes earlier in 16 out of 20 cells that overexpressed Spa2 (Fig 9B). The kinetics of actomyosin ring contraction were similar in control and cells overproducing Spa2. The average period of contraction was similar in both types of cells (a mean value of 6 min in control compared with 6.15 min in the *GAL-SPA2* cells). This finding would suggest that Spa2 promotes Chs2 incorporation at the site of division.

Since chitin synthase Chs2 promotes primary septum formation during cytokinesis, we investigated whether increased Spa2 protein can also induce higher levels of primary septum. Chs2 chitin synthase activity assays require the use of *chs3Δ* cells, as most of the chitin content is synthesised by chitin synthase Chs3 in budding yeast cells [47]. Cells were grown at 24°C and were synchronised in the G1 phase of the cell cycle. Then, we released cells from G1 block into medium containing calcofluor to stain primary septa and galactose to allow overexpression of Spa2. Progression through cytokinesis was similar in control and *GAL-SPA2* cells (Fig 9C (i)). To observe calcofluor-stained chitin in cells completing mitosis, cells were collected 135 minutes after release from G1 block when the percentage of cells containing primary septa peaks [14]. We showed that the relative signal intensity of primary septa was more than twice as strong in cells overexpressing Spa2 (Fig 9C (ii) (iii)), which support that Spa2 induces Chs2 incorporation. Taken together, these findings suggest that Spa2 has a direct role in recruiting the chitin synthase Chs2 to the site of division in budding yeast.

## Discussion

Our data highlight a key role for the cell polarity protein Spa2 during cytokinesis in budding yeast, and provide the first evidence of how specific factors contained within secretory vesicles, such as the protein Chs2, are incorporated into the cytokinetic machinery. Spa2 has previously been reported to form the so-called polarisome complex, which includes Pea2, Bud6, and the formin Bni1 [22, 23]. The polarisome functions in actin cytoskeletal organisation during polarised cell growth, which is important for numerous cellular functions including differentiation, proliferation, and morphogenesis [22, 23]. However, we found none of the other polarisome components in our purified material associated with Chs2-Inn1, suggesting an independent function during cytokinesis for Spa2.

**Fig 10:**
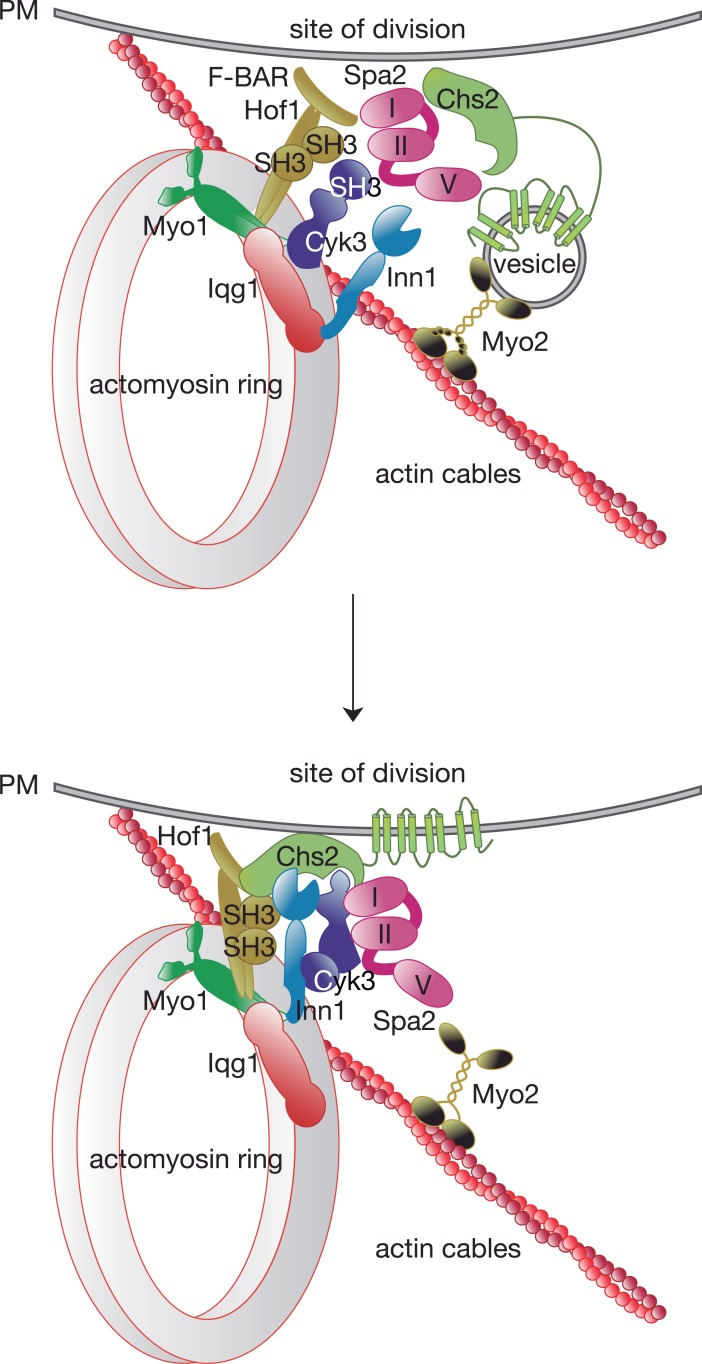
Spa2 protein coordinates specific delivery of secretory vesicles to the site of division during cytokinesis. Secretory vesicles are driven to the cleavage site by type V myosin-based transport. Spa2 interacts with the secretory vesicle system and IPC components Hof1 and Cyk3. We suggest that the three proteins coordinate the incorporation of chitin synthase Chs2 into the IPCs before the start of actomyosin ring contraction.

Spa2 directly interacts with IPC components during cytokinesis (Fig 10). Spa2 localisation requires the presence of an actomyosin ring and a functional secretory pathway contributes to Spa2 targeting at the division site. As has recently been described for the orthologue of Spa2 in *Candida albicans* [48], it seems very likely that kinase activity associated with mitotic CDK/Cyclin blocks Spa2 translocation to the cleavage site before chromosome segregation is resolved in *S*. *cerevisiae*, in a similar manner to that described for the assembly of the exocyst [49].

In *Xenopus* cells, disruption of the actomyosin ring blocks cleavage furrow formation, however the addition of membrane proceeds. This suggests that the actomyosin ring is important for restricting new membrane incorporation at the site of division [50–52]. Therefore, there must be a capture mechanism that allows the incorporation of transmembrane proteins transported on secretory vesicles into the actomyosin ring (Fig 10). We showed that the Spa2 protein might play such a role since it directly interacts with IPC components, Hof1 and Cyk3, and the chitin synthase Chs2. Spa2 could function as a bridge to allow specific Chs2 incorporation and membrane fusion of Chs2-containing vesicles at the site of division. It is of particular note that we found that increased levels of Spa2 promote Chs2 incorporation and primary septum formation. We have also previously shown that Inn1 regulates function and localisation of Chs2 [14, 44] and found that Inn1 plays a fundamental role in the incorporation of Chs2 at the cleavage site in the absence of Hof1 and Cyk3 (Fig 10). In addition, the C2 domain of Inn1 might also influence the fusion of Chs2-containing vesicles since the C2 domain of the human protein synaptotagmin is thought to contribute to the fusion of target membranes with synaptic vesicles [53].

The role of Spa2 protein during cytokinesis may well be conserved since *S*. *pombe* Spa2 was found to interact with Cdc15 [54], a Hof1 orthologue in fission yeast. Super-resolution microscopy and FRET techniques have recently revealed the nanoscale spatial organisation of fission yeast actomyosin ring components relative to the plasma membrane [55]. Spa2 is present in the same layer as proteins like Cyk3 and Fic1, the orthologue of budding yeast Inn1 [55]. Consistent with our results, Gould and colleagues were unable to find any association between other polarisome components and the cytokinetic machinery [54]. They found that Spa2 pulled down the Chs2 counterpart in fission yeast, the glycosyltransferase Bgs1 ((1,3)beta-D-glucan synthase catalytic subunit), which is required for primary septum formation [54, 56], although no molecular significance was noted. The Spa2-homology domain (SHD) is present in the mammalian GIT protein family, which is involved in cytoskeletal dynamics and membrane trafficking [34]. Thus, it seems that the role of the Spa2-homology domains in coordinating the assembly of larger complexes may be fundamental to modulating membrane trafficking and the targeting of specific cargoes to their intracellular destination.

## Methods

### Growth of yeast strains

The budding yeast *S*. *cerevisiae* strains used in this study were all based on W303 and are listed in S1 Table. Cells were grown in rich medium containing 1% yeast extract, 2% peptone and supplemented with 2% glucose (YPD), or 2% raffinose (YPRaff), or 2% galactose (YPGal). For all synchronisation experiments, asynchronous cultures of cells were grown overnight. The following morning cells were counted and diluted to a concentration of 4 x 10^6^ cells per ml before allowing them to grow to a density of 7 x 10^6^ cells per ml. To achieve synchrony of the yeast cultures we followed the previously described protocol [57]. To arrest cells in the G1 phase of the cell cycle, the mating pheromone α-factor (Pepceuticals Ltd) was added to a final concentration of 7.5 μg per ml. After 2 hours, additional 2.5 μg per ml aliquots of α-factor were added every 20 minutes and cells were checked using phase contrast microscopy until at least 90% of cells were unbudded. To release cells synchronously from G1 arrest, cells were pelleted, washed twice and released into fresh medium. Cells performing cytokinesis synchronously were collected 90 minutes after the release from alpha factor arrest when cells were grown in YPD medium at 24°C or 105 minutes when cells were grown in YPRaf at 24°C. On the other hand, cells were collected 75 minutes after the release from alpha factor arrest when cells were grown in YPGal medium at 37°C. Temperature and carbon source determine progression through the cell cycle. We arrested cells in the G2/M phase of the cell cycle by adding nocodazole to the medium at a final concentration of 5 μg per ml.

To stain primary septa of living cells, calcofluor was added 30 minutes after release from G1 block to a final concentration of 0.05 mg per ml and culture was incubated further for at least 60 minutes [58].

For time-lapse video microscopy in Fig 1D, cells were grown in YPD at 24°C, arrested in the G1 phase of the cell cycle. Cells were then released into YPD for 30 minutes before switching to Synthetic Complete (SC) medium to perform the time-lapse video microscopy. For time-lapse video microscopy depicted in Fig 8B, cells were grown in YPRaff at 24°C and arrested in G1 phase. Cells were then released into YPGal medium containing mating pheromone for 30 minutes before releasing cells from G1 block in YPGal medium for 75 minutes. Afterwards, cells were switched to SC medium in order to perform the time-lapse video microscopy.

In all experiments with temperature-sensitive degron strains (td), 0.1mM CuSO_4_ was included in the growth medium of exponential cultures before changing the carbon source to galactose to induce degradation [42]. To degrade proteins fused to the degron cassette, cells were transferred to YPGal medium at 24°C for 35 minutes to induce expression of *GAL-UBR1*, and then transferred to 37°C for 1 hour before release from the arrest [42]. For time-lapse video microscopy in S9C Fig, cells were grown in YPRaff at 24°C, arrested in G1 phase and degron protein depletion was achieved as described above. Subsequently, cells were released from G1 block into YPGal medium at 37°C for 30 minutes before switching to SC medium and placing them on the time-lapse slide to perform the time-lapse video microscopy. Cell cultures were maintained to monitor progression through the cell cycle for both strains under the same circumstances as time-lapse microscopy.

For experiments with *myo2-td* allele, cells were initially arrested in G1 phase using the mating pheromone α-factor. Subsequently, cells were released and blocked in early S phase of the cell cycle using hydroxyurea (Molekula Limited), which was added to a final concentration of 0.2 M. Cells were checked using phase contrast microscopy until at least 90% of cells contained a fully grown bud. At this point, Myo2-td inactivation was induced for 50 minutes, after which cells were released from hydroxyurea arrest.

### Analysis of cell growth on solid medium

Tenfold serial dilutions of fresh colonies of yeast cells were made and spots of cells containing between 50,000 and 50 cells were plated on the appropriate media. Plates were incubated for 2–3 days at the indicated temperature before the scan.

### Two-hybrid analysis

Two-hybrid analysis was performed using the vectors pGADT7 and pGBKT7 (Clontech). Cells were grown at 30°C on SC medium lacking leucine and tryptophan (non-selective) or lacking leucine, tryptophan and histidine (selective). They were scanned after 3–4 days of growth, as indicated.

### Expression and purification of recombinant proteins in *E*. *coli*

The plasmids to express recombinant proteins in *E*. *coli* used in this study were based on the ‘pET’ series (Novagen) and are listed in S2 Table. Recombinant proteins were expressed in Rosetta cell line at 37°C for 2 hours after induction with 1mM IPTG. Subsequently, pairs of cultures with induced proteins of choice were mixed so that each cell extract would contain two recombinant proteins. As control, a culture with an empty vector was mixed with the corresponding cultures expressing recombinant proteins. In all cases, after mixing, cell pellets were frozen at -20°C. To study the interaction between proteins, the strep-tagged fusions were isolated from a cell extract on 1 ml of Strep-Tactin Superflow resin (2-1206-025, IBA GmbH) before eluting with 2.5mM d-Desthiobiotin (D1411 Sigma). We detected the indicated proteins by immunoblotting with the previously described anti-StrepMAB Classic (2-1507-001, IBA GmbH) and Penta-His (34660, QIAGEN) antibodies.

### Immunoprecipitation of protein complexes from yeast cell extracts

To monitor the association of proteins in yeast cell extracts, we used 1000 ml samples (10^10^ cells). Frozen cell pellets were ground in the presence of liquid nitrogen, using a SPEX SamplePrep LLC 6850 freezer/mill as described previously [59]. We isolated tagged proteins by immunoprecipitating with magnetic Dynabeads M-270 Epoxy (Invitrogen) coupled at 4°C to rabbit anti-sheep IgGs (Sigma S-1265). We detected the indicated proteins by immunoblotting with the previously described polyclonal antibodies to Inn1, Chs2, Cyk3 and Hof1 [14], or by using polyclonal, anti-Spa2-yC-16 (Santa Cruz sc-15578), anti-FLAG antibody (Sigma F-7425), M2 anti-FLAG monoclonal antibody (Sigma F3165), peroxidase-antiperoxidase (PAP) (Sigma P1291), monoclonal 9E10 (anti-MYC) or 12CA5 (anti-HA).

### Mass spectrometry analysis

For mass spectrometry analysis of protein content, the digested peptides were analysed by nano LC/MS/MS with an ‘Orbitrap Velos’ (ThermoFisher). Data were processed as described previously (MS Bioworks) [14, 60, 61]. The total identification list was filtered at 1% FDR.

### Flow cytometry and binucleate cell analysis

We prepared samples to measure the DNA content or to determine the proportion of binucleate cells by fixing cells with 70% ethanol and staining with propidium iodide as described previously [57, 62]. Flow cytometry was performed with a Becton Dickinson FACSCanto II. For binucleate cell analysis, samples were then processed and images acquired with an upright fluorescence microscope (Axio Imager M1; Carl Zeiss, Inc.) using a 63x 0.95NA objective, an HRm camera, a Rhodamine specific filter set (em:546/12, exc: 608/65) and Axiovision software. We examined at least 100 cells at each time-point.

### Microscopy

Pictures of colonies on agar were taken after 24 hours (YPD medium) or 30 hours (YPGal medium) with a Nikon CoolPix 995 camera attached to a Nikon Eclipse E400 microscope. To observe GFP-tagged proteins cells were fixed with 8% formaldehyde for 10 minutes and subsequently washed twice with ice-cold PBS. Phase contrast and fluorescence microscopy images of cells grown in liquid culture were performed with a Nikon A1R Microscope and an Orca R2 camera (Hamamatsu) with objective lens Plan Apo TIRF 100x oil DIC 1.49NA, and LightLine single-band filter set FITC Semrock. The illumination source was a Nikon Intensilight C-HGFIE (ultrahigh presure 130W mercury lamp). We used NIS elements software. We analysed eleven z-sections with a spacing of 0.375 μm to facilitate the examination of whole cells for all experiments. Exposure time, sensor gain and digital adjustments were the same for control and experimental samples. We examined 100 cells for each time-point. Each experiment was carried out at least three times.

For time-lapse video microscopy cells were grown in an IBIDI cells in focus 15 μ -slide (8 well glass bottom; 80827) after their release from G1 arrest. The base of each well is formed of a glass coverslip that we coated with a 5 mg per ml solution of the lectin Concanavalin A (Sigma L7647), and then washed with water and dried for 30 minutes. A suspension of cells was then placed on the glass coverslip and incubated for 5 min in order to allow cells to attach. The coverslip was then washed with pre-warmed SC medium. Finally 300 μl pre-warmed SC medium was added. Time-lapse video microscopy illustrated in Fig 1D was performed using the DeltaVision system with an Olympus IX-71 microscope and a CoolSNAP HQ2 Monochrome camera. A Plapon 60X0 1.42 NA objective lens was used. The 300W xenon system with liquid light guide was used for illumination. Images were captured with Softworx Resolve 3D acquisition software. We analysed 8 z-sections with a spacing of 0.4 μm. For time-lapse video microscopy shown in Fig 9B and S9C Fig, 9 z-sections with a spacing of 0.4 μm were acquired with a widefield epifluorescence microscope (Nikon Eclipse Ti2) equipped with an APO TIRF x100/1.49 objective and an sCMOS camera (Hamamatsu Orca-Flash4.0). Brightfield and fluorescence images were sequentially acquired every 2 minutes for 1 hour at 24ºC or 37ºC. Focus drift was avoided with the help of a hardware-based focusing system (Nikon´s Perfect Focus System).

The microscopy data were deconvolved using Huygens (SVI) according to the “Quick Maximum Likelihood Estimation” method and a measured point spread function. The deconvolved data set was viewed with “ImageJ” software (National Institute of Health, USA) [63]

## Supporting information

S1 Fig **(A)** Tetrad analysis of the meiotic progeny from the indicated diploid strain (YMF708) shows the synthetic lethality of *spa2Δ cyk3Δ* cells. Spores of the indicated genotypes were grown for 20 hours on YPD plates at 24°C. Scale bar: 20 μm (i). Tetrad analysis of the meiotic progeny from the indicated diploid strains (YMF1664) (ii) and YMF1667 (iii)) shows the synthetic lethality between *spa2Δ* and *cyk3-2A-ΔSH3* cells. ‘UG‘ denotes *ungerminated spore*. **(B)** Tetrad analysis of the meiotic progeny from the indicated diploid strain (YMF168) shows the synthetic lethality of *spa2Δ hof1Δ* cells. Spores of the indicated genotypes were grown for 20 hours on YPD plates at 24°C. Scale bar: 20 μm (i). **(C)** Tetrad analysis of the meiotic progeny from the indicated diploid strains: (i) YMF837, (ii) YMF824 and (iii) YMF1261 shows the synthetic lethality of *spa2Δ hof1-ΔFBAR-ΔSH3* cells.
(EPS)

S2 Fig *SPA2-GFP* (YMF167) and *SPA2-GFP iqg1-td* (YMF183) strains were arrested in G1 phase at 24°C in YPRaff and then shifted to YPGal at 37°C to deplete Iqg1-td. Subsequently, cells were released to allow progression through the cell cycle and DNA content was measured by flow cytometry. Examples of cells are shown for specific time-points. Scale bar: 10 μm.
(EPS)

S3 Fig **(A)** *SPA2-GFP* (YMF167) and *SPA2-GFP myo1-td* (YMF185) strains were arrested in G1 phase at 24°C in YPRaff and then shifted to YPGal at 37°C to deplete Myo1-td. Subsequently, cells were released to allow progression through the cell cycle. Samples were taken at the indicated times to determine the proportion of binucleate cells (i) and the percentage of cells with single Spa2 rings at the cleavage site (ii). **(B)** *SPA2-GFP* (YMF167) and *SPA2-GFP inn1-td* (YMF164) strains were grown as in (A). Subsequently, cells were released to allow progression through the cell cycle. Samples were taken at the indicated times to determine the proportion of binucleate cells (i) and the percentage of cells with single Spa2 rings at the cleavage site (ii).
(EPS)

S4 Fig **(A)** *SPA2-GFP cyk3-td* strain (YMF1104) was grown in YPRaff at 24°C in YPRaff and then shifted to YPGal at 37°C to determine Cyk3 protein stability. Samples were taken as indicated and protein extracts prepared before immunoblotting with anti-Cyk3 antibodies. **(B)** *SPA2-GFP myo2-td* (YMF716) strain was grown in YPRaff at 24°C in YPRaff and then shifted to YPGal at 37°C to study Myo2 protein levels at restrictive conditions. Samples were taken as indicated and protein extracts prepared before immunoblotting with anti-DHFR antibodies.
(EPS)

S5 Fig **(A)** *SEC8-GFP* (YMF872) and *SEC8-GFP iqg1-td* (YMF1432) strains were arrested in G1 phase at 24°C in YPRaff and then shifted to YPGal at 37°C to deplete Iqg1-td. Subsequently, cells were released to allow progression through the cell cycle. Samples were taken at the indicated times to determine the proportion of binucleate cells (i) and the percentage of cells with single Sec8 rings at the cleavage site (ii). **(B)** *SEC8-GFP* (YMF872) and *SEC8-GFP hof1-td* (YMF909) strains were grown in YPRaff as in (A) and cells were then released to allow progression through the cell cycle. Samples were taken at the indicated times to determine the proportion of binucleate cells (i) and the percentage of cells with Sec8 rings at the cleavage site (ii).
(EPS)

S6 Fig *CHS2-GFP* (YMF330) and *CHS2-GFP myo2-td* (YMF869) strains were arrested in G1 phase at 24°C in YPRaff and then synchronously shifted to YPRaff medium containing 0.2 M hydroxyurea. Therefore cells were arrested at the early S phase, but bud growth continued. Cells were then transferred to YPGal containing 0.2 M hydroxyurea at 37°C in order to deplete Myo2-td. Subsequently, cells were released to allow progression through the cell cycle. Samples were taken at the indicated times to determine the proportion of binucleate cells (i) and the percentage of cells with Chs2 rings at the cleavage site (ii).
(EPS)

S7 Fig *SPA2-GFP* (YMF167) and *SPA2-GFP hof1-td myo2-td* (YMF1418) strains were arrested in G1 phase at 24°C in YPRaff and then synchronously shifted to YPRaff medium containing 0.2 M hydroxyurea and arrested in early S phase. Before cells were transferred to YPGal containing 0.2 M hydroxyurea at 37°C in order to deplete Hof1-td and Myo2-td, they were allowed to grow their buds. Subsequently, cells were released to allow progression through the cell cycle and DNA content was measured by flow cytometry. Examples of cells are shown for specific time-point. Scale bar: 10 μm.
(EPS)

S8 Fig **(A)** Examples of cells depicted in Fig 5D are shown with Spa2-GFP single ring at the bud-neck at 90 minutes for Spa2-GFP and 105 minutes for Spa2-1-552-GFP and Spa2-553-1466-GFP. Scale bar: 2 μm. **(B)** *SPA2-GFP* (YMF117), *SPA2-1-552-GFP* (YMF967) and *SPA2-553-1466-GFP* (YMF1023) strains were grown asynchronously at 24°C in YPD. Samples to monitor the level of corresponding proteins expressed under the control of *SPA2* promoter were collected. Protein extracts were prepared before immunoblotting with anti-GFP antibodies. As protein levels varied, two different exposure times are presented.
(EPS)

S9 Fig **(A)** *SPA2-GFP* (YMF167) and *SPA2-GFP hof1-td cyk3-td* (YMF1088) strains were arrested in G1 phase at 24°C in YPRaff and then shifted to YPGal at 37°C to deplete Hof1-td and Cyk3-td simultaneously. Subsequently, cells were released to allow progression through the cell cycle and samples were taken and protein extracts prepared before immunoblotting with anti-GFP antibodies to detect Spa2 protein level. **(B)** *CHS2-GFP* (YMF330) and *CHS2-GFP hof1-td cyk3-td* (YMF1076) strains were grown in YPRaff as in (A). Samples were taken at the indicated times to determine the level of Chs2 protein using anti-Chs2 antibodies. **(C)** *CHS2-GFP SPC42-EQFP* (YAD380) and *CHS2-GFP hof1-td cyk3-td* (YMF1076) strains were arrested in G1 phase at 24°C in YPRaff and then shifted to YPGal at 37°C to deplete Hof1-td and Cyk3-td proteins simultaneously. Subsequently, cells were released for 30 minutes in YPGal at 37°C to allow progression through the cell cycle. An aliquot of cells was then transferred to SC medium and placed on the time-lapse slide to examine the localisation of Chs2 every 2 minutes as cells completed cell division at 37°C (see Methods for details). A z-stack of images was gathered. A two-dimensional projection of the three-dimensional data is shown. Scale bar: 2 μm. The grey and black circles denote the timing of the actomyosin ring contraction for control cells (i). To confirm cell cycle progression, cell cultures from which aliquots were taken to perform time-lapse microscopy were kept growing under the same experimental conditions. The proportion of binucleate cells is shown in (ii).
(EPS)

S1 Table Strains used in this study (all based on W303). (DOC)

S2 Table Plasmids used to express recombinant proteins in *E*. *coli*. (DOC)
