# Supplementary material for: Cell polarity protein Spa2 coordinates Chs2 incorporation at the division site in budding yeast
Source: PLoS Genet. 2018 Mar 30;14(3):e1007299. doi: 10.1371/journal.pgen.1007299 (PMC5895073; doi:10.1371/journal.pgen.1007299)
Supplement: S1 Table — (DOC) [file pgen.1007299.s010.doc]

| **Strain Name** | **Genotype** | **Sourc*e*** |
| --- | --- | --- |
| W303-1 | *MAT***a /** *MATα*  *ade2-1 / ade2-1 ura3-1 / ura3-1 his3-11,15 / his3-11,15 trp1-1 / trp1-1 leu2-3,112 / leu2-3,112 can1-100 / can1-100* | R. Rothstein |
| W303-1a | *MAT***a** *ade2-1 ura3-1 his3-11,15 trp1-1 leu2-3,112 can1-100* | R. Rothstein |
| YMF10 | *MAT***a** *ade2-1 ura3-1 his3-11,15 trp1-1 leu2-3,112 can1-100*  *pep4∆::URA3 (URA3) ADE2* | This study |
| YMF38 | *MAT***a** *ade2-1 ura3-1 his3-11,15 trp1-1 leu2-3,112 can1-100*  *CHS2-9MYC (K.l.TRP1) INN1-TAP (kanMX) pep4∆::URA3 (URA3) ADE2* | This study |
| YMF79 | *MAT***a** *ade2-1 ura3-1 his3-11,15 trp1-1 leu2-3,112 can1-100*  *CHS2-9MYC (K.l.TRP1) leu2-3,112::TETO2-CTAP4 (LEU2) pep4∆::URA3 (URA3) ADE2* | This study |
| YMF117 | *MAT***a** *ade2-1 ura3-1 his3-11,15 trp1-1 leu2-3,112 can1-100*  *SPA2-GFP (K.l.TRP1)* | This study |
| YMF140 | *MAT***a** *ade2-1 ura3-1 his3-11,15 trp1-1 leu2-3,112 can1-100*  *ubr1∆::GAL-HA-UBR1 (HIS3) cyk3-td (K.l.TRP1) hof1-td (hphNT)* | This study |
| YMF164 | *MAT***a** *ade2-1 ura3-1 his3-11,15 trp1-1 leu2-3,112 can1-100*  *ubr1∆::GAL-HA-UBR1 (HIS3) SPA2-GFP (K.l.TRP1) inn1-td (kanMX)* | This study |
| YMF167 | *MAT***a** *ade2-1 ura3-1 his3-11,15 trp1-1 leu2-3,112 can1-100*  *ubr1∆::GAL-HA-UBR1 (HIS3) SPA2-GFP (K.l.TRP1)* | This study |
| YMF168 | *MAT***a /** *MAT***α** *ade2-1 / ade2-1 ura3-1 / ura3-1 his3-11,15 / his3-11,15 trp1-1 / trp1-1 leu2-3,112 / leu2-3,112 can1-100 / can1-100 SPA2 / spa2∆ (HIS3MX) HOF1 / hof1∆ (hphNT)* | This study |
| YMF183 | *MAT***a** *ade2-1 ura3-1 his3-11,15 trp1-1 leu2-3,112 can1-100*  *ubr1∆::GAL-HA-UBR1 (HIS3) SPA2-GFP (K.l.TRP1) iqg1-td (hphNT)* | This study |
| YMF185 | *MAT***a** *ade2-1 ura3-1 his3-11,15 trp1-1 leu2-3,112 can1-100*  *ubr1∆::GAL-HA-UBR1 (HIS3) SPA2-GFP (K.l.TRP1) myo1-td (hphNT)* | This study |
| YMF330 | *MAT***a** *ade2-1 ura3-1 his3-11,15 trp1-1 leu2-3,112 can1-100*  *ubr1∆::GAL-HA-UBR1 (HIS3) CHS2-GFP (K.l.TRP1)* | This study |
| YMF505 | *MAT***a** *ade2-1 ura3-1 his3-11,15 trp1-1 leu2-3,112 can1-100*  *chs3∆ (URA3)* | This study |
| YMF708 | *MAT***a /** *MAT***α** *ade2-1 / ade2-1 ura3-1 / ura3-1 his3-11,15 / his3-11,15 trp1-1 / trp1-1 leu2-3,112 / leu2-3,112 can1-100 / can1-100 SPA2 / spa2∆ (HIS3MX) CYK3 / cyk3∆ (hphNT)* | This study |
| YMF713 | *MAT***a** *ade2-1 ura3-1 his3-11,15 trp1-1 leu2-3,112 can1-100*  *ubr1∆::GAL-HA-UBR1 (HIS3) hof1-td (hphNT) SPA2-GFP (K.l.TRP1)* | This study |
| YMF716 | *MAT***a** *ade2-1 ura3-1 his3-11,15 trp1-1 leu2-3,112 can1-100*  *ubr1∆::GAL-HA-UBR1 (HIS3) SPA2-GFP (K.l.TRP1) myo2-td (hphNT)* | This study |
| YMF741 | *MAT***a /** *MATα* *ade2-1 / ade2-1 ura3-1 / ura3-1 his3-11,15 / his3-11,15 trp1-1 / trp1-1 leu2-3,112 / leu2-3,112 can1-100 / can1-100 SPA2 / spa2∆ (HIS3MX) CYK3 / cyk3∆ (K.l.TRP1) CHS2 / CHS2-V377I (hphNT)* | This study |
| YMF759 | *MAT***a** *ade2-1 ura3-1 his3-11,15 trp1-1 leu2-3,112 can1-100*  *ubr1∆::GAL-HA-UBR1 (HIS3) spa2∆ (HIS3MX)* | This study |
| YMF824 | *MAT***a /** *MAT***α** *ade2-1 / ade2-1 ura3-1 / ura3-1 his3-11,15 / his3-11,15 trp1-1 / trp1-1 leu2-3,112 / leu2-3,112 can1-100 / can1-100 SPA2 / spa2∆ (HIS3MX) HOF1 / hof1∆sh3 (kanMX)* | This study |
| YMF837 | *MAT***a /** *MAT***α** *ade2-1 / ade2-1 ura3-1 / ura3-1 his3-11,15 / his3-11,15 trp1-1 / trp1-1 leu2-3,112 / leu2-3,112 can1-100 / can1-100 SPA2 / spa2∆ (HIS3MX) HOF1 / hof1 ∆fbar (kanMX)* | This study |
| YMF866 | *MAT***a /** *MATα* *ade2-1 / ade2-1 ura3-1 / ura3-1 his3-11,15 / his3-11,15 trp1-1 / trp1-1 leu2-3,112 / leu2-3,112 can1-100 / can1-100 SPA2 / spa2∆ (HIS3MX) HOF1 / hof1∆ (K.l.TRP1) CHS2 / CHS2-V377I (hphNT)* | This study |
| YMF869 | *MAT***a** *ade2-1 ura3-1 his3-11,15 trp1-1 leu2-3,112 can1-100*  *ubr1∆::GAL-HA-UBR1 (HIS3) CHS2-GFP (K.l.TRP1) myo2-td (hphNT)* | This study |
| YMF872 | *MAT***a** *ade2-1 ura3-1 his3-11,15 trp1-1 leu2-3,112 can1-100*  *ubr1∆::GAL-HA-UBR1 (HIS3) SEC8-GFP (K.l.TRP1)* | This study |
| YMF909 | *MAT***a** *ade2-1 ura3-1 his3-11,15 trp1-1 leu2-3,112 can1-100*  *ubr1∆::GAL-HA-UBR1 (HIS3) SEC8-GFP (K.l.TRP1) hof1-td (hphNT)* | This study |
| YMF914 | *MAT***a** *ade2-1 ura3-1 his3-11,15 trp1-1 leu2-3,112 can1-100*  *MYO2-5FLAG (URA3CP) pep4∆::ADE2 (ADE2)* | This study |
| YMF967 | *MAT***a** *ade2-1 ura3-1 his3-11,15 trp1-1 leu2-3,112 can1-100*  *spa2∆-553-1466-GFP (kanMX)* | This study |
| YMF969 | *MAT***a** *ade2-1 ura3-1 his3-11,15 trp1-1 leu2-3,112 can1-100*  *MYO2-5FLAG (URA3CP) pep4∆::ADE2 (ADE2) INN1-TAP (kanMX)* | This study |
| YMF1023 | *MAT***a** *ade2-1 ura3-1 his3-11,15 trp1-1 leu2-3,112 can1-100*  *spa2∆-1-552-GFP (kanMX, K.l.TRP1)* | This study |
| YMF1076 | *MAT***a** *ade2-1 ura3-1 his3-11,15 trp1-1 leu2-3,112 can1-100*  *ubr1∆::GAL-HA-UBR1 (HIS3) cyk3-td (K.l.TRP1) hof1-td (hphNT) CHS2-GFP (K.l.TRP1)* | This study |
| YMF1088 | *MAT***a** *ade2-1 ura3-1 his3-11,15 trp1-1 leu2-3,112 can1-100*  *ubr1∆::GAL-HA-UBR1 (HIS3) cyk3-td (K.l.TRP1) hof1-td (hphNT) SPA2-GFP (K.l.TRP1)* | This study |
| YMF1104 | *MAT***a** *ade2-1 ura3-1 his3-11,15 trp1-1 leu2-3,112 can1-100*  *ubr1∆::GAL-HA-UBR1 (HIS3) cyk3-td (K.l.TRP1) SPA2-GFP (K.l.TRP1)* | This study |
| YMF1176 | *MAT***a** *ade2-1 ura3-1 his3-11,15 trp1-1 leu2-3,112 can1-100*  *MYO1-5FLAG (hphNT) pep4∆::URA3 (URA3) NTAP-SPA2 (kanMX) IQG1-6HA (K.l.TRP1) ADE2* | This study |
| YMF1178 | *MAT***a** *ade2-1 ura3-1 his3-11,15 trp1-1 leu2-3,112 can1-100*  *MYO1-5FLAG (hphNT) pep4∆::URA3 (URA3) IQG1-6HA (K.l.TRP1) ADE2* | This study |
| YMF1256 | *MAT***a** *ade2-1 ura3-1 his3-11,15 trp1-1 leu2-3,112 can1-100*  *SPA2-GFP (K.l.TRP1) MYO1-Tomatoe (kanMX)* | This study |
| YMF1261 | *MAT***a /** *MAT***α** *ade2-1 / ade2-1 ura3-1 / ura3-1 his3-11,15 / his3-11,15 trp1-1 / trp1-1 leu2-3,112 / leu2-3,112 can1-100 / can1-100 SPA2 / spa2∆ (HIS3MX) HOF1 / hof1∆sh3∆fbar (kanMX, URA3)* | This study |
| YMF1268 | *MAT***a** *ade2-1 ura3-1 his3-11,15 trp1-1 leu2-3,112 can1-100*  *ubr1∆::GAL-HA-UBR1 (HIS3) hof1-td (hphNT)* | This study |
| YMF1299 | *MAT***a** *ade2-1 ura3-1 his3-11,15 trp1-1 leu2-3,112 can1-100*  *MYO2-5FLAG (URA3CP) pep4∆::ADE2 (ADE2) NTAP-SPA2 (kanMX) CHS2-9MYC (K.l.TRP1) ubr1∆::GAL-HA-UBR1 (HIS3) hof1-td (hphNT)* | This study |
| YMF1301 | *MAT***a** *ade2-1 ura3-1 his3-11,15 trp1-1 leu2-3,112 can1-100*  *MYO2-5FLAG (URA3CP) pep4∆::ADE2 (ADE2) NTAP-SPA2 (kanMX) CHS2-9MYC (K.l.TRP1) ubr1∆::GAL-HA-UBR1 (HIS3)* | This study |
| YMF1302 | *MAT***a** *ade2-1 ura3-1 his3-11,15 trp1-1 leu2-3,112 can1-100*  *pep4∆::ADE2 (ADE2) NTAP-SPA2 (kanMX) CHS2-9MYC (K.l.TRP1)* | This study |
| YMF1307 | *MAT***a** *ade2-1 ura3-1 his3-11,15 trp1-1 leu2-3,112 can1-100*  *ubr1∆::GAL-HA-UBR1 (HIS3) cyk3-td (K.l.TRP1) hof1-td (hphNT) CHS2-V377I (hphNT)* | This study |
| YMF1329 | *MAT***a** *ade2-1 ura3-1 his3-11,15 trp1-1 leu2-3,112 can1-100*  *ubr1∆::GAL-HA-UBR1 (HIS3) cyk3-td (K.l.TRP1) hof1-td (hphNT) CHS2-V377I-GFP (K.l.TRP1)* | This study |
| YMF1357 | *MAT***a** *ade2-1 ura3-1 his3-11,15 trp1-1 leu2-3,112 can1-100*  *ubr1∆::GAL-HA-UBR1 (HIS3) hof1-td (hphNT) spa2∆ (HIS3MX)* | This study |
| YMF1370 | *MAT***a** *ade2-1 ura3-1 his3-11,15 trp1-1 leu2-3,112 can1-100*  *ubr1∆::GAL-HA-UBR1 (HIS3) cyk3-td (K.l.TRP1) hof1-td (hphNT) inn1-td (kanMX) CHS2-V377I-GFP (K.l.TRP1)* | This study |
| YMF1375 | *MAT***a** *ade2-1 ura3-1 his3-11,15 trp1-1 leu2-3,112 can1-100*  *ubr1∆::GAL-HA-UBR1 (HIS3) inn1-td (kanMX) CHS2-V377I-GFP (K.l.TRP1)* | This study |
| YMF1394 | *MAT***a** *ade2-1 ura3-1 his3-11,15 trp1-1 leu2-3,112 can1-100*  *ubr1∆::GAL-HA-UBR1 (HIS3) cyk3-td (K.l.TRP1) hof1-td (hphNT) inn1-td (kanMX)* | This study |
| YMF1399 | *MAT***a** *ade2-1 ura3-1 his3-11,15 trp1-1 leu2-3,112 can1-100*  *ubr1∆::GAL-HA-UBR1 (HIS3) cyk3-td (K.l.TRP1) hof1-td (hphNT) spa2∆ (HIS3MX) CHS2-V377I (hphNT)* | This study |
| YMF1401 | *MAT***a** *ade2-1 ura3-1 his3-11,15 trp1-1 leu2-3,112 can1-100*  *ubr1∆::GAL-HA-UBR1 (HIS3) cyk3-td (K.l.TRP1) hof1-td (hphNT) spa2∆ (HIS3MX)* | This study |
| YMF1403 | *MAT***a** *ade2-1 ura3-1 his3-11,15 trp1-1 leu2-3,112 can1-100*  *ubr1∆::GAL-HA-UBR1 (HIS3) cyk3-td (K.l.TRP1) hof1-td (hphNT) SPA2-GFP (K.l.TRP1) CHS2-V377I (hphNT)* | This study |
| YMF1418 | *MAT***a** *ade2-1 ura3-1 his3-11,15 trp1-1 leu2-3,112 can1-100*  *ubr1∆::GAL-HA-UBR1 (HIS3) SPA2-GFP (K.l.TRP1) myo2-td (hphNT) hof1-td (hphNT)* | This study |
| YMF1432 | *MAT***a** *ade2-1 ura3-1 his3-11,15 trp1-1 leu2-3,112 can1-100*  *ubr1∆::GAL-HA-UBR1 (HIS3) SEC8-GFP (K.l.TRP1) iqg1-td (hphNT)* | This study |
| YMF1448 | *MAT***a /** *MATα* *ade2-1 / ade2-1 ura3-1 / ura3-1 his3-11,15 / his3-11,15 trp1-1 / trp1-1 leu2-3,112 / leu2-3,112 can1-100 / can1-100 SPA2-5FLAG (HIS3MX) / NTAP-SPA2 (kanMX) pep4∆::ADE2 (ADE2) / pep4∆::URA3 (URA3) ADE2 / ade2-1* | This study |
| YMF1449 | *MAT***a /** *MATα* *ade2-1 / ade2-1 ura3-1 / ura3-1 his3-11,15 / his3-11,15 trp1-1 / trp1-1 leu2-3,112 / leu2-3,112 can1-100 / can1-100 SPA2-5FLAG (HIS3MX) / SPA2 pep4∆::ADE2 (ADE2) / pep4∆::URA3 (URA3) ADE2 / ade2-1* | This study |
| YMF1534 | *MAT***a** *ade2-1 ura3-1 his3-11,15 trp1-1 leu2-3,112 can1-100*  *chs3∆ (URA3) GAL-SPA2 (LEU2)* | This study |
| YMF1660 | *MAT***a** *ade2-1 ura3-1 his3-11,15 trp1-1 leu2-3,112 can1-100*  *CHS2-GFP (K.l.TRP1) GAL-SPA2 (LEU2)* | This study |
| YMF1664 | *MAT***a /** *MAT***α** *ade2-1 / ade2-1 ura3-1 / ura3-1 his3-11,15 / his3-11,15 trp1-1 / trp1-1 leu2-3,112 / leu2-3,112 can1-100 / can1-100 SPA2 / spa2∆ (HIS3MX) cyk3-2A (hphNT) / sh3∆cyk3 (kanMX)* | This study |
| YMF1667 | *MAT***a /** *MAT***α** *ade2-1 / ade2-1 ura3-1 / ura3-1 his3-11,15 / his3-11,15 trp1-1 / trp1-1 leu2-3,112 / leu2-3,112 can1-100 / can1-100 SPA2 / spa2∆ (HIS3MX) CYK3 / sh3∆cyk3-2A (kanMX, hphNT)* | This study |
| YASD522 | *MAT***a** *ade2-1 ura3-1 his3-11,15 trp1-1 leu2-3,112 can1-100*  *ubr1∆::GAL-HA-UBR1 (HIS3) inn1-td (kanMX)* | This study |
| YASD819 | *MAT***a** *ade2-1 ura3-1 his3-11,15 trp1-1 leu2-3,112 can1-100*  *CHS2-GFP (K.l.TRP1)* | This study |
| YAD380 | *MAT***a** *ade2-1 ura3-1 his3-11,15 trp1-1 leu2-3,112 can1-100*  *CHS2-GFP (K.l.TRP1) SPC42-EQFP (HIS3MX)* | This study |
