# Supplementary material for: Cell polarity protein Spa2 coordinates Chs2 incorporation at the division site in budding yeast
Source: PLoS Genet. 2018 Mar 30;14(3):e1007299. doi: 10.1371/journal.pgen.1007299 (PMC5895073; doi:10.1371/journal.pgen.1007299)
Supplement: S2 Table — (DOC) [file pgen.1007299.s011.doc]

| **Plasmid** | **Expressed proteins** |
| --- | --- |
| pMF30 | 6His-inn1 |
| pMF36 | Strep-tag-Chs2-215-629 |
| pMF49 | Strep-tag-Chs2-215-629-V377I |
| pMF62 | 6His-Cyk3-475-885 |
| pMF104 | 6His-Spa2-1-552 |
| pMF111 | Strep-tag-Hof1-1-300 |
| pMF116 | Strep-tag -Spa2-1-552 |
| pASD41 | Strep-tag -SH3-Hof1 |
| pASD44 | Strep-tag -SH3-Cyk3 |
